# From “Undetectable” to “Sensitive Detection”: Advances in Derivatization Techniques for LC-MS Analysis of Genotoxic Impurities

**DOI:** 10.3390/molecules31162889

**Published:** 2026-08-19

**Authors:** Xingchen Wang, Zhuzi Chen, Shunli Ji

**Affiliations:** 1 College of Food and Biotechnology, Wuhu Vocational Technical University, Wuhu 241000, China; 2 Office of Scientific Research, Jiangsu Health Vocational College, Nanjing 211800, China; 3 Department of Pharmaceutical Analysis, China Pharmaceutical University, Nanjing 210009, China

**Keywords:** genotoxic impurities, derivatization, LC-MS/MS, sample pretreatment, ICH M7

## Abstract

Many genotoxic impurities (GTIs) remain “invisible” to conventional LC-MS due to poor ionization or chemical instability under electrospray ionization, yet their sub-ppm acceptable intake limits under ICH M7(R2) demand exceptional analytical sensitivity. Derivatization—the chemical introduction of ionizable moieties, stable tags, or MS/MS information carriers—offers a powerful strategy to overcome this limitation. This review provides a critical systematic overview of derivatization techniques for LC-MS analysis of GTIs over the past decade (2015–2025, based on a literature search across PubMed, Web of Science, and Scopus). We construct a functional-group-based strategic framework covering alkyl halides, nitroaromatics, sulfonyl chlorides, hydroxylamine, alcohols, aldehydes, carboxylic acids, and amines, while placing specific emphasis on typical impurities within these classes such as methyl iodide, methyl chloride, nitrobenzene, and benzenesulfonyl chloride, and discuss the evolution of reagents from simple “reaction tags” to “MS/MS information carriers” that provide characteristic neutral losses or product ions for enhanced selectivity. Quantitative analysis reveals that derivatization typically enhances ESI response by 2–3 orders of magnitude, consistently achieving LODs below 1 ppm—the ICH M7(R2) threshold. Key analytical trade-offs are critically evaluated, including the balance between derivatization efficiency and reaction time, by-product management, and the fundamental kinetic and chromatographic constraints that render post-column derivatization impractical for most GTIs. We conclude with perspectives on high-throughput automation, smart multifunctional reagents, online integration, and green chemistry, aiming to provide a practical roadmap for developing robust, sensitive, and regulatory-compliant LC-MS methods for GTI control.

## 1. Introduction

Genotoxic impurities (GTIs) are DNA-reactive substances that pose a carcinogenic risk even at trace levels [1,2,3,4,5]. Structurally, most GTIs contain electrophilic functional groups—such as alkyl halides, epoxides, nitroaromatics, and aldehydes—that can covalently bind to DNA [6,7]. Unlike conventional impurities with threshold effects, GTIs are considered non-threshold toxicants; the ICH M7(R2) guideline therefore sets an acceptable intake of 1.5 μg per person per day, which translates to sub-ppm concentration limits in active pharmaceutical ingredients (APIs).

Liquid chromatography-tandem mass spectrometry (LC-MS/MS) is the workhorse for trace impurity analysis due to its high sensitivity and selectivity [8,9,10,11,12]. However, when applied to GTIs, LC-MS faces two fundamental bottlenecks. First, many GTIs are neutral, small-molecule electrophiles that lack basic or acidic functional groups, rendering them poorly ionizable in electrospray ionization (ESI) [13,14]. Second, complex API matrices often cause severe ion suppression or enhancement, compromising quantitative accuracy even with multiple reaction monitoring [3,15]. As a result, a significant number of GTIs remain effectively “invisible” to conventional LC-MS, creating an urgent need for alternative analytical strategies [15,16].

**Figure 1:**
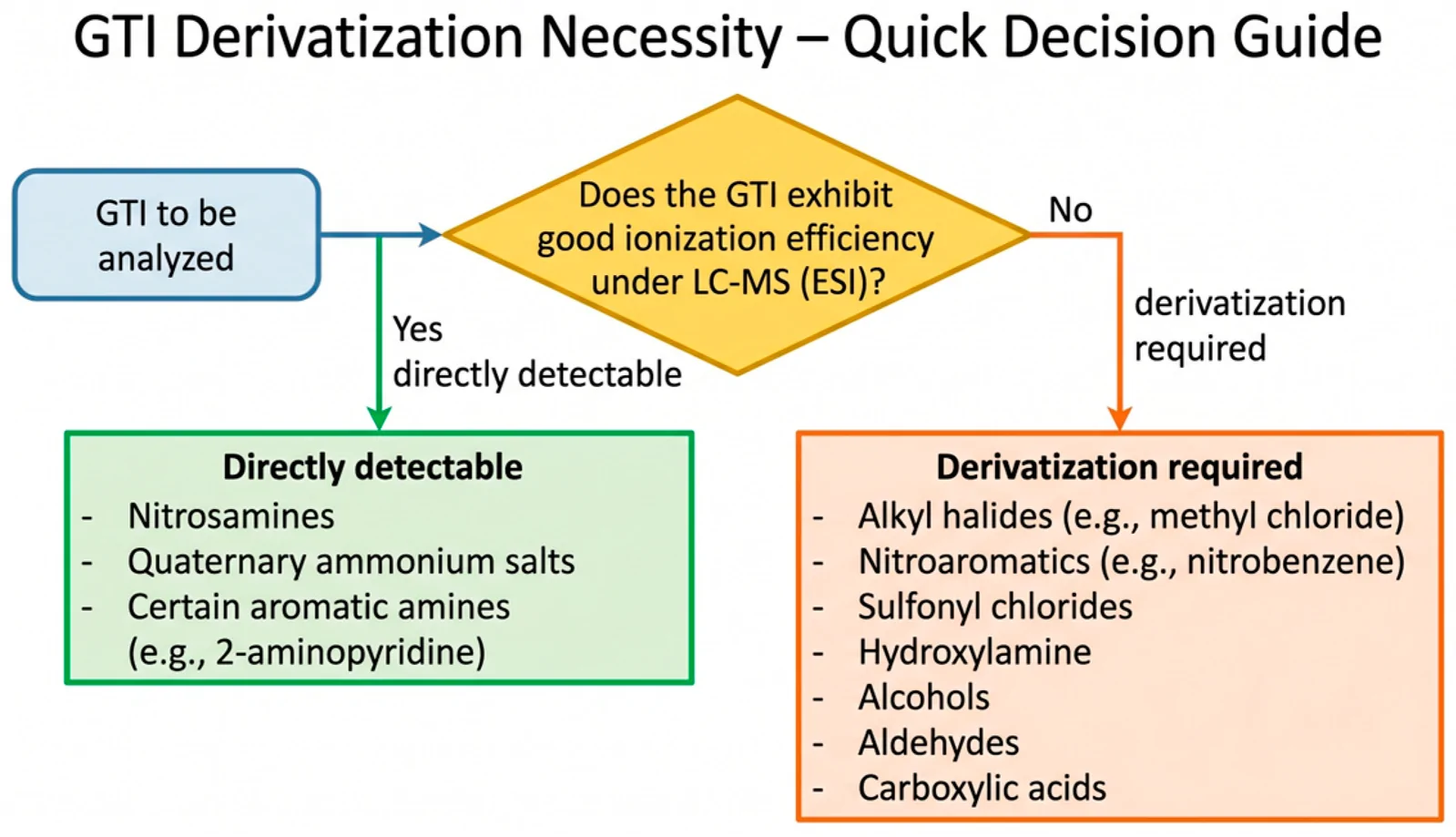
GTI derivatization necessity—quick decision guide. The flowchart guides analysts to decide whether derivatization is required based on the GTI’s ionization efficiency under ESI-MS and its functional group.

Derivatization—chemical modification of the target analyte to introduce ionizable moieties, improve chromatographic retention, or embed characteristic mass spectrometric tags—offers a straightforward and powerful solution to these bottlenecks [14,17]. In the context of GTI analysis, derivatization provides four core benefits: (i) it dramatically enhances ESI response by adding permanent charges or protonatable groups; (ii) it improves reversed-phase retention by adjusting analyte polarity; (iii) it enables selective MS/MS filtering (e.g., neutral loss scanning) through rationally designed reagent side chains that yield characteristic fragmentation patterns; and (iv) when combined with isotope labeling, it corrects for matrix effects and yield fluctuations [18]. Across different GTI classes, derivatization has been shown to improve ESI-MS sensitivity by factors ranging from 50 to over 1000, with most robust methods achieving LODs at or below the sub-ppm level required for routine quality control. To help analysts quickly determine whether a specific GTI requires derivatization under typical LC-ESI-MS conditions, Figure 1 presents a straightforward decision flowchart based on the analyte’s intrinsic ionization efficiency and chemical class. Depending on the GTI’s functional group, a variety of derivatization chemistries have been established, including nucleophilic substitution (e.g., for alkyl halides), reduction (for nitroaromatics), acylation (for hydroxylamine and amines), and esterification (for alcohols). These strategies can be implemented in pre-column or post-column modes, each with distinct trade-offs in reaction kinetics, automation potential, and chromatographic resolution [19,20].

Raman et al. have previously reviewed derivatization-LC-MS methods for genotoxic impurities, covering alkyl sulfonates, hydrazines, aldehydes, and other functional groups with validation data [21]. Despite the proliferation of derivatization methods for GTIs over the past decade, the literature remains fragmented. Most published reports focus on a single impurity class or a specific reagent, leaving analysts without a systematic framework to navigate the key decisions: Which derivatization reagent should be chosen for a given GTI functional group? How to balance reactivity, selectivity, and MS compatibility? What are the acceptable conversion yield and reproducibility for regulatory compliance? Moreover, critical issues such as by-product interference, matrix effects on the derivatization reaction itself, and the frequent failure of post-column approaches are often underemphasized [22,23,24].

This review provides a critical and systematic overview of derivatization techniques for LC-MS analysis of GTIs. We first outline the regulatory landscape established by the ICH M7(R2) guideline and the fundamental chemical principles that make derivatization indispensable for the trace analysis of mutagenic impurities. We then construct a functional-group-based strategic framework, covering alkyl halides, nitroaromatics, sulfonyl chlorides, hydroxylamine, alcohols, aldehydes, carboxylic acids, and amines. The design philosophy of derivatization reagents is discussed, emphasizing the evolution from simple “reaction tags” to “MS/MS information carriers” that provide characteristic neutral losses or product ions for enhanced selectivity. Key analytical trade-offs—derivatization efficiency, by-product control, and the practical unsuitability of post-column derivatization for most GTIs—are critically evaluated. We also compare different derivatization reagents and discuss pre-column versus post-column modes. Finally, we offer perspectives on high-throughput automation, smart reagent design (multi-tag, cleavable, stimuli-responsive), online integration, and green chemistry. It is important to note that not all GTIs require derivatization. Nitrosamines, for example, are generally detectable by conventional LC-MS/MS due to their good ionization efficiency under ESI^+^ or APCI^+^; moreover, they have been extensively reviewed elsewhere [25]. Therefore, they are not covered in this review. The goal of this review is to act as a hands-on reference for pharmaceutical analysts in the development of LC-MS methods that are robust, sensitive, and fully compliant with regulatory requirements for GTI control.

## 2. Fundamental Principles and Strategic Framework of Derivatization in GTI Analysis

### 2.1. Chemical Basis of Derivatization: From Functional Group Reactivity to MS Detectability

Most GTIs are small, electrophilic molecules that contain structural alert groups such as alkyl halides, epoxides, nitroaromatics, sulfonyl chlorides, and aldehydes [26]. While these electrophilic centers are responsible for DNA reactivity, they also render the analytes neutral and devoid of basic or acidic functional groups. Under electrospray ionization (ESI), such compounds exhibit extremely poor ionization efficiency, making them effectively “invisible” to conventional LC-MS [13,27].

Derivatization overcomes this limitation by chemically converting the GTI into a derivative that carries a permanent charge or a readily protonatable/deprotonatable group, thereby enhancing ESI response by two to three orders of magnitude [28]. Four general derivatization strategies are relevant to GTI analysis, each exploiting the intrinsic reactivity of the target functional group. The critical role of derivatization in improving chromatographic retention, eliminating carryover, and forming stable derivatives for MS/MS analysis has been systematically reviewed in the context of veterinary drug residue analysis, providing a useful reference for GTI method development [29].

Nucleophilic substitution (SN_2_) is the most direct approach for alkyl halides and related electrophiles that contain a good leaving group [3]. A nucleophile attacks the electrophilic carbon, displacing the leaving group and forming a quaternary ammonium salt, which gives an intense signal in ESI^+^ mode. The reaction rate follows the well-established order: iodides > bromides >> chlorides [30]. This trend dictates that derivatization of alkyl chlorides often requires more forcing conditions (higher temperature, longer time, or stronger nucleophiles). For less reactive substrates such as alkyl chlorides, more powerful nucleophiles or extended reaction times are required.

Reductive derivatization is necessary for GTIs that lack a leaving group, such as nitroaromatics. Reduction of the nitro group to a primary amine yields a species that is readily protonated under acidic LC conditions [31]. Various reducing systems have been employed, including metal-acid combinations (e.g., zinc dust/ammonium formate), catalytic hydrogenation (Pd/C/H2), and sodium dithionite. Each system offers distinct trade-offs in terms of reaction speed, selectivity, and compatibility with API functional groups.

Stabilization derivatization addresses chemically unstable GTIs [3]. For example, sulfonyl chlorides hydrolyze rapidly in the presence of trace moisture. Derivatization with a suitable amine (e.g., benzylamine) “locks” the reactive center as a stable sulfonamide, simultaneously introducing a protonatable group for ESI detection [23]. This principle extends to any reactive GTI that degrades under standard analytical conditions; derivatization must therefore occur immediately upon sample preparation.

Elemental tagging offers an alternative when ESI-based methods fail. Introduction of a heteroatom (e.g., iodine, bromine) via acylation or alkylation allows detection by inductively coupled plasma-mass spectrometry (ICP-MS). This approach bypasses organic matrix effects entirely but requires specialized instrumentation not commonly found in quality control laboratories [30].

The choice among these strategies is dictated by the GTI’s functional group, the API’s tolerance to reaction conditions, and the required sensitivity. However, conceptually feasible derivatization does not guarantee a reliable analytical method. The following sections establish a systematic framework for evaluating the critical performance metrics that determine method robustness and regulatory compliance.

### 2.2. Critical Analytical Performance Metrics and Regulatory Alignment

**Figure 2:**
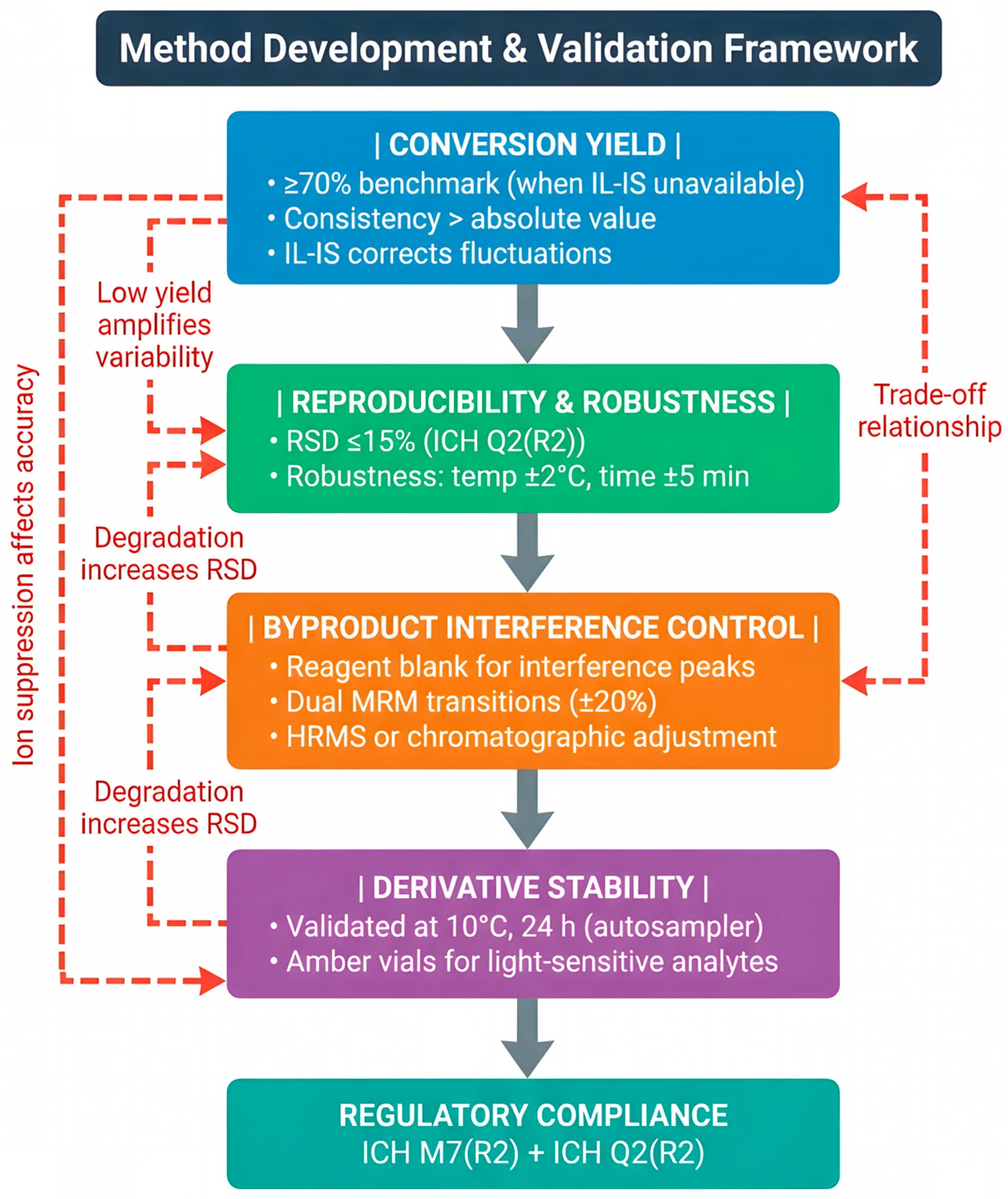
Four Critical Analytical Performance Metrics for Derivatization-Based Genotoxic Impurity (GTI) Methods. Stepwise validation framework for derivatization-based GTI methods. Dashed arrows on the left indicate cross-influences among metrics.

The reliability of any derivatization-based method for GTI analysis hinges on four interrelated metrics: conversion yield, reproducibility (including robustness), byproduct interference, and derivative stability [21]. These metrics are not merely academic—they are directly linked to the expectations of ICH M7(R2) (acceptable intake of 1.5 μg per person per day) and ICH Q2(R2) (validation of analytical procedures). For a drug product with a maximum daily dose of 1 g, this intake limit translates to a permissible concentration of approximately 1.5 ppm in the active pharmaceutical ingredient (API). Therefore, any analytical method intended for GTI control must demonstrate a limit of quantification (LOQ) at or below this threshold in the actual API matrix [24,32]. Figure 2 provides a conceptual overview of these four metrics and their interrelationships within a stepwise validation framework.

#### 2.2.1. Conversion Yield and Its Regulatory Significance

Conversion yield is defined as the percentage of the target GTI transformed into the derivative under defined reaction conditions. It should be distinguished from “recovery,” which combines derivatization yield, extraction efficiency, and matrix effects. Two approaches are recommended for yield determination. The first is direct comparison of the derivative peak area against a pure synthetic standard. The second is the “difference method,” which measures residual underivatized GTI. A survey of published GTI derivatization methods reveals that fewer than half report such yield data, and even fewer use a pure derivative standard [33].

What constitutes an acceptable conversion yield? While neither ICH M7(R2) nor any other regulatory guideline prescribes a specific numerical threshold for derivatization efficiency, we advocate a practical benchmark of ≥70% for quantitative GTI methods intended for routine quality control. This recommendation is substantiated by several interrelated considerations. Primarily, under ICH Q2(R2), the precision at the LOQ is expected to be within ≤15% RSD. Our experience, corroborated by systematic observations in method development, indicates that when conversion yields fall below 70%, the inherent sensitivity of the derivatization kinetics to minor experimental fluctuations—such as temperature variations of ±2 °C, time deviations of ±5 min, or batch-to-batch reagent differences—frequently translates to yield variations that exceed this acceptable precision window, particularly in the absence of an isotopically labeled internal standard (IL-IS). Furthermore, in the absence of an IL-IS, maintaining a yield of ≥70% provides a crucial safety margin, ensuring that the total method variability (encompassing derivatization, extraction, and injection) remains within the ±15% acceptance criteria widely adopted for trace-level impurity quantification [33,34]. This 70% benchmark also aligns well with the conventional recovery ranges (typically 80–120%) that are considered acceptable in spike-recovery validation studies for genotoxic impurities, as recommended by ICH M7(R2). Moreover, empirical evidence from the authors’ cross-project method transfer experiences—spanning alkyl halides, nitroaromatics, and sulfonyl chlorides—reveals that methods operating below this threshold are disproportionately susceptible to inter-laboratory failure unless an IL-IS is employed. It is important to note, however, that this threshold is not absolute; when a rigorously validated IL-IS that undergoes the same derivatization reaction as the analyte is available, a lower conversion yield may be tolerable, provided that the consistency of the reaction (RSD ≤5%) is unequivocally demonstrated across the intended concentration range.

What constitutes an acceptable conversion yield? While no universal threshold exists, a yield of ≥70% is proposed here as a practical guideline for quantitative methods intended for routine quality control, based on the authors’ experience in method development and transfer across multiple GTI projects. This recommendation is grounded in the observation that below 70%, small variations in reaction conditions (temperature ±2 °C, time ±5 min, reagent lot) can cause disproportionately large changes in yield [34]. Critically, because ICH M7 requires quantification at the sub-ppm level, any yield fluctuation directly impacts the ability to reliably detect concentrations at the acceptable intake limit. A yield of ≥70% provides a reasonable safety margin against such fluctuations. However, it is critical to recognize that the absolute yield value is less important than its consistency. A derivatization that achieves only 50% conversion but remains stable within ±5% RSD across all samples, standards, and quality control materials—especially when an isotopically labeled internal standard (IL-IS) is employed—can be more reliable than one that yields 90% but fluctuates between 70% and 95%. In the absence of an IL-IS, stricter control of reaction parameters and a higher yield (≥70%) become necessary to ensure quantification accuracy at the sub-ppm level mandated by ICH M7(R2).

The most effective approach to mitigate yield fluctuations is the use of an isotopically labeled internal standard (IL-IS) that undergoes the same derivatization reaction. Because the IL-IS experiences identical yield variations, the analyte/IS peak area ratio cancels out fluctuations [35]. When an IL-IS is unavailable, a structural analog that reacts similarly can be used, albeit with lower precision. In the absence of any internal standard, rigorous control of reaction parameters and inclusion of a derivatized control standard in every batch become mandatory.

#### 2.2.2. Reproducibility, Robustness and ICH Q2(R2) Expectations

Reproducibility is typically reported as the relative standard deviation (RSD%) of replicate preparations. For trace GTI analysis, acceptance criteria should follow general bioanalytical guidelines: within-run RSD ≤15% and between-run RSD ≤15% at the LOQ level. However, inter-day and inter-analyst reproducibility are rarely shown in published GTI derivatization methods. A method that passes validation with RSD <5% in the hands of the original developer may fail during transfer to another site due to differences in solvent brand, ambient temperature, or operator technique—a challenge well documented in inter-laboratory studies of LC-MS methods [36].

Robustness testing—deliberately varying critical parameters (e.g., solvent brand, temperature ±5 °C, reaction time ±10%) to identify factors that significantly affect yield—is explicitly recommended by ICH Q2(R2). Any method that fails robustness testing is not fit for routine use. Common pitfalls include the presence of trace water or peroxides in the solvent, which can oxidize certain derivatization reagents or hydrolyze reactive intermediates. Freshly opened anhydrous solvents and storage of reagents under inert atmosphere are strongly recommended.

#### 2.2.3. Byproduct Interference and Ion Suppression—Avoiding False Results

Byproducts can arise from self-reactions of the derivatization reagent (e.g., dimerization) or from side reactions with solvents or matrix components [37]. These byproducts pose three types of analytical interference: (i) co-elution with the target derivative, leading to inaccurate peak integration; (ii) ion suppression or enhancement due to high background signals; and (iii) false-positive signals if the byproduct shares the same MRM transitions as the derivative [38,39]. This type of interference is not merely a chromatographic nuisance; it can compromise the analytical result through two distinct mechanisms. First, if the byproduct co-elutes with the target derivative, it competes for droplet charges in the ESI source, causing ion suppression that reduces the analyte signal [38]. Second, if the byproduct shares precursor or product ions with the derivative (as observed for certain DMAP dimers and their quaternized products), it can generate false-positive MRM signals, particularly when the derivative is present at trace levels near the LOQ [40]. To distinguish between true analyte signals and byproduct-related artifacts, the analyst should: (i) include a reagent blank in every batch to identify byproduct peaks; (ii) monitor two complementary MRM transitions for the derivative and verify that their ion ratio falls within ±20% of the theoretical value; and (iii) when co-elution is unavoidable, consider using high-resolution mass spectrometry (HRMS) or adjusting chromatographic conditions to achieve baseline separation. Because the LOQ for GTIs is often near the lower limit of the mass spectrometer’s dynamic range, even modest ion suppression can push the effective LOQ above the ICH M7 limit, rendering the method invalid for its intended purpose.

A reagent blank chromatogram should be included in every validation to identify byproduct peaks. Optimization of reaction conditions (lower temperature, shorter time) to suppress byproduct formation remains the first line of defense. Beyond byproduct interference, ion suppression caused by the derivatization reagent, its byproducts, or co-eluting matrix components must be quantitatively assessed. A recommended approach is post-column infusion of the derivatized blank (i.e., reagent blank after derivatization) while injecting the analyte standard; any dip in the analyte signal at the retention time of the derivative indicates suppression from the reagent matrix [38]. Conversely, infusion of the analyte while injecting the derivatized blank reveals suppression caused by the reagent components. This method allows direct localization and quantification of suppression effects, typically reported as the percentage signal loss relative to a neat standard [41].

#### 2.2.4. Derivative Stability

Derivative stability must be assessed under anticipated storage conditions, typically in the autosampler at 10 °C for 24 h [42]. Some derivatives (e.g., certain quaternary ammonium salts) show gradual degradation, leading to decreasing peak areas over a run sequence [43]. If significant instability is observed, alternative storage conditions (e.g., lower temperature, amber vials for light-sensitive derivatives) or re-derivatization before each injection may be required. Method validation reports should explicitly state the established stability window; failure to do so risks rejection by regulatory authorities or failed sample batches due to time-dependent signal loss.

#### 2.2.5. Matrix Effects on Derivatization Efficiency

A critical but often overlooked aspect is the assessment of API matrix effects on derivatization efficiency. A simple post-extraction spike recovery experiment—where the GTI is spiked into the final sample extract—is insufficient because it does not reveal whether the API itself competes for the derivatization reagent or reducing agent during the reaction step [22,30,44]. To address this, a two-tiered approach is recommended. First, the derivatization yield of the GTI in neat solution should be compared with that in the presence of the API at its target concentration. If the recovery deviates by more than ±15% from the neat-solution value, further investigation is warranted. Second, a “derivatization robustness” study should be performed by varying the API concentration (e.g., 50–150% of nominal) while keeping the GTI spike constant. If the measured GTI level changes systematically with API concentration, matrix interference is confirmed. Mitigation strategies include increasing the molar excess of the derivatization reagent, introducing a selective cleanup step (such as solid-phase extraction) before derivatization, or switching to an alternative derivatization chemistry that is less susceptible to competition [34,45].

#### 2.2.6. The Role of Isotopically Labeled Internal Standards in Regulatory Compliance

One of the most debated topics in GTI method development is the necessity of an isotopically labeled internal standard (IL-IS). ICH M7(R2) does not explicitly mandate an IL-IS for every GTI method, but the guideline strongly emphasizes accuracy and precision at trace levels. Given that derivatization yields are notoriously variable due to fluctuations in reaction temperature, time, reagent quality, and matrix composition, an IL-IS that undergoes the same derivatization reaction as the analyte provides the most robust correction for yield variations and matrix effects. This principle is well established in isotope dilution mass spectrometry [46]. However, for many GTIs, a suitable IL-IS is not commercially available or is prohibitively expensive. In such cases, a structural analog that reacts similarly can be used as a surrogate internal standard, provided its equivalence is rigorously validated—for example, by demonstrating parallel dilution curves across the expected concentration range. When no internal standard of any kind is available, the method must rely on extremely tight control of reaction parameters (temperature, time, reagent concentration) and the inclusion of a derivatized control standard in every analytical batch, with stricter acceptance criteria for recovery (typically 85–115%).

### 2.3. Trade-Offs in Derivatization Method Development

Developing a derivatization-based method for GTI analysis inevitably involves balancing competing objectives, and recognizing these trade-offs is essential for rational method design. No single derivatization protocol can simultaneously maximize reaction speed, conversion yield, selectivity, and sensitivity while minimizing byproduct formation and instrumentation requirements. Instead, the optimal method represents a context-dependent compromise that must be explicitly evaluated during development and documented in validation reports.

Reaction speed versus substrate scope. Derivatization reactions that proceed to completion within 30 min under mild conditions are generally effective for highly reactive GTIs, such as alkyl iodides and bromides, which possess good leaving groups and readily undergo SN_2_ substitution [42]. However, extending the same method to less reactive analogues—most notably alkyl chlorides—often requires dramatically extended reaction times, ranging from several hours to a full day, even with elevated temperatures and more powerful nucleophiles [42]. Such prolonged reaction times may be impractical for routine batch release testing, where analytical turnaround time is a critical operational parameter. Conversely, accepting a lower conversion yield for chlorides in exchange for a shorter reaction time risks inadequate sensitivity, as the reduced amount of derivative may fail to meet the required LOQ mandated by ICH M7. This trade-off forces the analyst to decide whether the method must cover a broad range of alkyl halides (including slow-reacting chlorides) or only the specific, fast-reacting impurities expected in a given synthetic route.

Conversion yield versus byproduct formation. Increasing the reaction temperature or the concentration of the derivatization reagent generally improves the yield of the desired derivative by shifting the equilibrium or accelerating the reaction kinetics. However, these same conditions often promote side reactions—most notably, dimerization, oligomerization, or degradation of the reagent itself. For example, certain tertiary amine-based derivatization reagents have been reported to form dimeric byproducts at elevated temperatures, generating chromatographic peaks that may co-elute with the target derivative and cause either integration errors or ion suppression [42]. Similarly, excess reagent can react with adventitious water or solvent impurities, producing a high chemical background that elevates baseline noise. The optimal reaction conditions therefore represent a compromise in which the yield is sufficient to achieve the required LOQ in the API matrix, while byproduct peaks are either chromatographically resolved from the derivative or, if co-elution is unavoidable, do not produce interfering MRM transitions or cause unacceptable ion suppression [38].

Selectivity versus universality of the derivatization reagent. Highly selective reagents—those designed to react with a specific functional group (e.g., alkyl halides) while showing minimal cross-reactivity with other moieties—offer the advantage of simpler sample preparation and fewer interference peaks. However, selectivity comes at the cost of a priori knowledge: the analyst must know or suspect which GTI functional group is present. Such reagents cannot be used for untargeted screening of unknown impurities. Conversely, universal reagents that react with a broad range of nucleophiles—such as 9-fluorenylmethyl chloroformate (FMOC-Cl), which reacts with primary amines, secondary amines, hydroxylamine, and even water—provide wide applicability across multiple GTI classes in a single workflow [42]. The price of this universality is poor selectivity, which often necessitates extensive sample cleanup (e.g., API precipitation, solid-phase extraction) to remove nucleophilic components of the API and matrix that would otherwise consume the reagent and generate interfering peaks. The choice between selective and universal reagents thus depends critically on the analytical context: targeted quantification of a known GTI versus screening for multiple potential impurities.

Sensitivity versus instrumentation accessibility. For the vast majority of GTIs, conventional ESI-MS coupled with pre-column derivatization using commercially available reagents provides a widely accessible, cost-effective platform that can achieve sub-ppm sensitivity when properly optimized [20,47]. However, a small subset of GTIs remains intractable even after extensive tagging efforts—for example, certain aliphatic alcohols or hydrazines that do not ionize efficiently even as derivatives. In such cases, elemental tagging with ICP-MS offers a powerful alternative, achieving sub-ppb detection limits by introducing a heteroatom (e.g., iodine, bromine) and detecting it with the near-universal, matrix-independent response of ICP-MS. This exceptional sensitivity comes at the cost of specialized instrumentation not routinely available in pharmaceutical QC laboratories, limited compatibility with organic mobile phases (typically requiring ≤20–40% acetonitrile with oxygen addition to maintain plasma stability), and higher per-sample operating expenses. Elemental tagging should therefore be reserved for those cases where all ESI-compatible approaches have been exhausted and the regulatory limit cannot otherwise be met.

Summary of trade-offs. In summary, no universally “best” derivatization method exists. The optimal choice is always context-dependent, guided by the specific GTI’s reactivity, the API’s tolerance to reaction conditions, the required LOQ, and the available instrumentation. These trade-offs must be explicitly evaluated and documented during method development and validation, as they directly influence the robustness and regulatory acceptability of the final method.

## 3. Derivatization Reagents: Design and Case Studies

Once the fundamental principles and performance metrics of derivatization are understood, the analyst must select the most appropriate reagent for a given GTI and analytical context. This chapter provides a systematic comparison of derivatization reagents across different functional groups, followed by a case study on the evolution from simple reaction tags to MS/MS information carriers, using the DMAP-to-BPPC transition as an illustrative example.

### 3.1. Cross-Comparison of Derivatization Reagents by Functional Group

No single derivatization reagent is optimal for all GTIs. Table 1 summarizes representative derivatization methods for a range of functional group types, including alkyl halides, nitroaromatics, sulfonyl chlorides, hydroxylamine, alcohols, hydrazines, azides, carbamates, aziridines, pyridinium salts, aromatic amines, aldehydes, and carboxylic acids. For each entry, the table provides the derivatization reagent, reaction conditions, detection method, limit of detection (LOD), recovery, precision (RSD), and reference. It should be noted that direct cross-comparison is limited by differences in API matrices and instrumentation across studies.

Several trends emerge from Table 1. For electrophilic GTIs with high reactivity—alkyl halides and sulfonyl chlorides—derivatization strategies are almost exclusively based on nucleophilic substitution. For example, methyl iodide is converted to a quaternary ammonium salt with DMAP in 30 min with >95% conversion [30], whereas the much less reactive methyl chloride requires a more powerful reagent (BPPC) and 24 h to reach 40–50% conversion [42]. Similarly, benzenesulfonyl chloride is locked with benzylamine to form a stable sulfonamide [23]. These reactions typically proceed at 50–60 °C, but the required reaction time varies by orders of magnitude—from minutes to a full day—reflecting the intrinsic electrophilicity of the target GTI.

For GTIs that lack an electrophilic centre—nitroaromatics, hydroxylamine, and hydrazines—direct nucleophilic attack is not possible, so alternative strategies are required. Nitrobenzene is reduced to aniline using zinc dust/ammonium formate [31]; hydroxylamine is derivatized with FMOC-Cl [42]; and phenylhydrazine is converted with 2,3,5-triiodobenzoyl chloride [42]. These transformations are generally mild (room temperature to 60 °C) and fast (30–60 min), but selectivity can be an issue: the reducing agent may also attack other reducible groups in the API, and FMOC-Cl reacts with any nucleophile present.

A completely different philosophy is elemental tagging. 4-Chloro-1-butanol is esterified with 3-iodobenzoyl chloride, and the iodine tag is detected by LC-ICP-MS with sub-ppm sensitivity [42]. This approach bypasses ESI-related matrix effects entirely but requires an ICP-MS instrument, which is not standard in most QC laboratories.

Table 1 also lists several GTIs that can be detected directly without derivatization, such as the azide [42], the pyridinium salt 4-dimethylaminopyridine [34], and the aromatic amine 2-aminopyridine. Their intrinsic ionizability under ESI conditions makes derivatization unnecessary, reminding analysts that the first step in method development should always be to evaluate the MS response of the target GTI itself. To enable direct cross-comparison of analytical performance across the diverse studies compiled in Table 1, all limit of detection (LOD) values have been uniformly converted to parts per million (ppm, μg·g^−1^) under the following assumptions. For absolute mass-based LODs (e.g., 50 pg injection for hexanal), an injection volume of 10 μL was assumed to calculate the corresponding concentration. For molar-based LODs (e.g., 25 nM for valproic acid), conversion employed the respective molecular weight (144.2 g/mol for valproic acid). For solution-based values reported in μg·L^−1^ (e.g., 3.5 μg·L^−1^ for carbaryl), conversion assumed a typical final sample concentration of 1 mg·mL^−1^. The ‘Sensitivity Enhancement’ column provides conservative estimates of the fold-increase in detectability achieved by derivatization, based on direct comparison with the underivatized analyte under comparable LC-MS conditions as reported in the original references. Where such data were not available, ‘NE’ (not estimated) is indicated. Values not reported in the original reference are marked ‘NR’ (not reported). Recovery and relative standard deviation (RSD) values are presented as originally published; cross-study comparisons should consider differences in active pharmaceutical ingredient (API) matrices, spiking levels, and instrumentation.

The methods compiled in Table 1 and discussed above span a wide spectrum of derivatization chemistries. When evaluated collectively, several overarching trade-offs emerge. For highly reactive electrophiles such as alkyl iodides, bromides, and sulfonyl chlorides, fast nucleophilic capture (e.g., with DMAP or benzylamine) affords high conversion (>95%) and short reaction times (30 min), making these reagents the first choice for targeted quality control methods. In contrast, less reactive substrates such as alkyl chlorides require more powerful reagents (e.g., BPPC), which enable selective MS/MS detection via neutral-loss scanning but demand prolonged reaction times (24 h) and yield only moderate conversion (40–50%)—a trade-off that is acceptable only when an isotopically labeled internal standard is employed. For nitroaromatic GTIs, reductive derivatization with Zn/NH_4_COOH provides mild and fast (30 min) conversion, yet the reducing agent may attack other reducible functionalities in the API, necessitating case-by-case selectivity assessment. Hydroxylamine derivatization with FMOC-Cl is effective for polar analytes that lack chromatographic retention, but the reagent’s non-selectivity toward any nucleophile often requires prior API removal via precipitation. Finally, elemental tagging with ICP-MS, while capable of bypassing ESI matrix effects entirely, remains a specialized fallback due to instrumentation requirements and limited organic mobile phase compatibility. These comparisons illustrate that no single method is universally optimal; the choice must be guided by the GTI’s intrinsic reactivity, the API’s functional group tolerance, the required sensitivity, and the available instrumentation.

### 3.2. From Reaction Tags to MS/MS Information Carriers: The DMAP-to-BPPC Evolution

Among all entries in Table 1, the evolution from DMAP to BPPC for alkyl halides best illustrates how reagent engineering can address reactivity and selectivity limitations while embedding mass spectrometric information into the reagent structure.

DMAP (4-dimethylaminopyridine) is a highly nucleophilic tertiary amine that reacts with alkyl halides via an SN_2_ mechanism to form a permanently charged quaternary ammonium salt. Under optimized conditions (60 °C, 30 min, acetonitrile), DMAP converts alkyl iodides and bromides with >95% conversion, enabling LC-MS/MS detection limits below 1 ppm [30]. However, DMAP is ineffective for alkyl chlorides, achieving <5% conversion under the same conditions due to the poor leaving-group ability of chloride.

To overcome this limitation, van Wijk and co-workers designed BPPC (butyl 1-(pyridin-4-yl)piperidine-4-carboxylate), a reagent that retains the pyridine-based nucleophile but incorporates a butyl ester side chain [42]. The structural modifications serve two purposes. First, the increased basicity and steric accessibility of the piperidine nitrogen enhance the reactivity toward less electrophilic alkyl chlorides. Second, the butyl ester side chain undergoes preferential fragmentation under collision-induced dissociation (CID), yielding a characteristic neutral loss of 56 Da (corresponding to butene or butanol elimination). This allows neutral loss scanning (NLS) as a selective detection mode, effectively eliminating false positives from co-eluting matrix components.

The trade-offs are substantial. BPPC achieves only 40–50% conversion for methyl chloride after 24 h at 60 °C in the presence of 1% ammonia (to maintain high pH). The method is therefore better suited for screening applications where the presence of alkyl chlorides is suspected, rather than for precise quantification without an internal standard. For routine targeted analysis of known alkyl iodides or bromides, DMAP remains the preferred choice due to its speed and high conversion yield.

**Figure 3:**
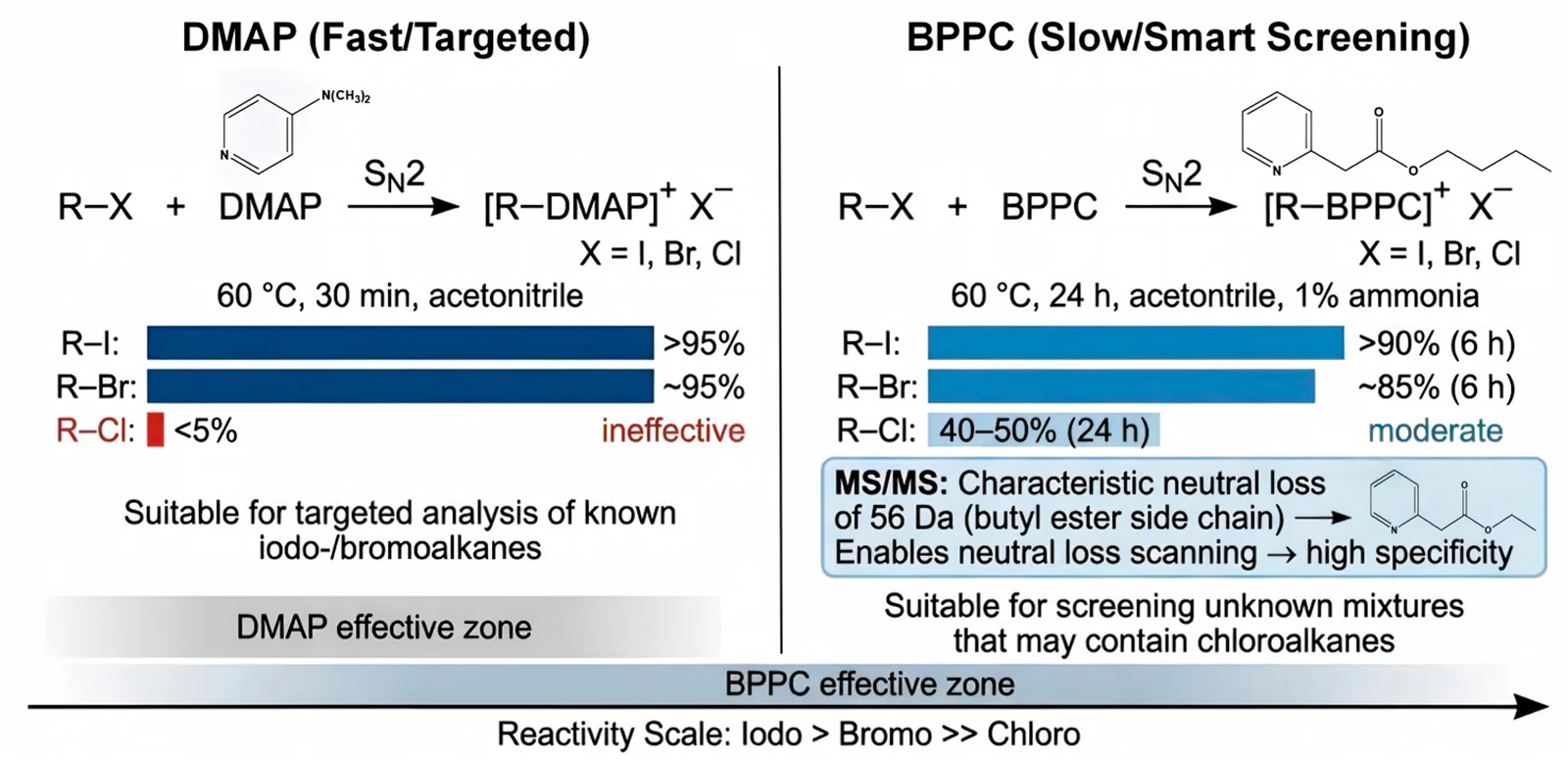
Comparison of Derivatization Strategies for Alkyl Halide GTIs: DMAP (Fast/Targeted) vs. BPPC (Slow/Smart Screening).

The DMAP-to-BPPC journey illustrates a broader design paradigm: derivatization reagents can be engineered not only to improve reactivity but also to “write” MS/MS information (characteristic neutral losses or product ions) into the derivative. This concept can be generalized. For example, pentafluorobenzylhydroxylamine (PFBHA) used for aldehyde derivatization provides excellent response in electron capture negative ionization MS, and its characteristic fragmentation enables selective detection [55]. Similarly, hexyl chloroformate derivatization of aromatic amines increases hydrophobicity and improves reversed-phase retention while introducing a fragmentable carbamate linkage [45]. The contrasting characteristics of DMAP and BPPC—in terms of reaction speed, conversion yield, substrate scope, and detection mode—are summarized in Figure 3.

Looking forward, the design of derivatization reagents is evolving from experience-driven to strategy-driven. Incorporating isotopically labeled tags, cleavable linkers, or stimuli-responsive moieties are promising directions for next-generation reagents. However, the fundamental principle remains unchanged: the reagent must be matched to the GTI’s functional group, the API’s tolerance, and the analytical question at hand.

## 4. Derivatization Strategies for Different Functional Groups: Practical Challenges and Solutions

### 4.1. Alkyl Halides

**Figure 4:**
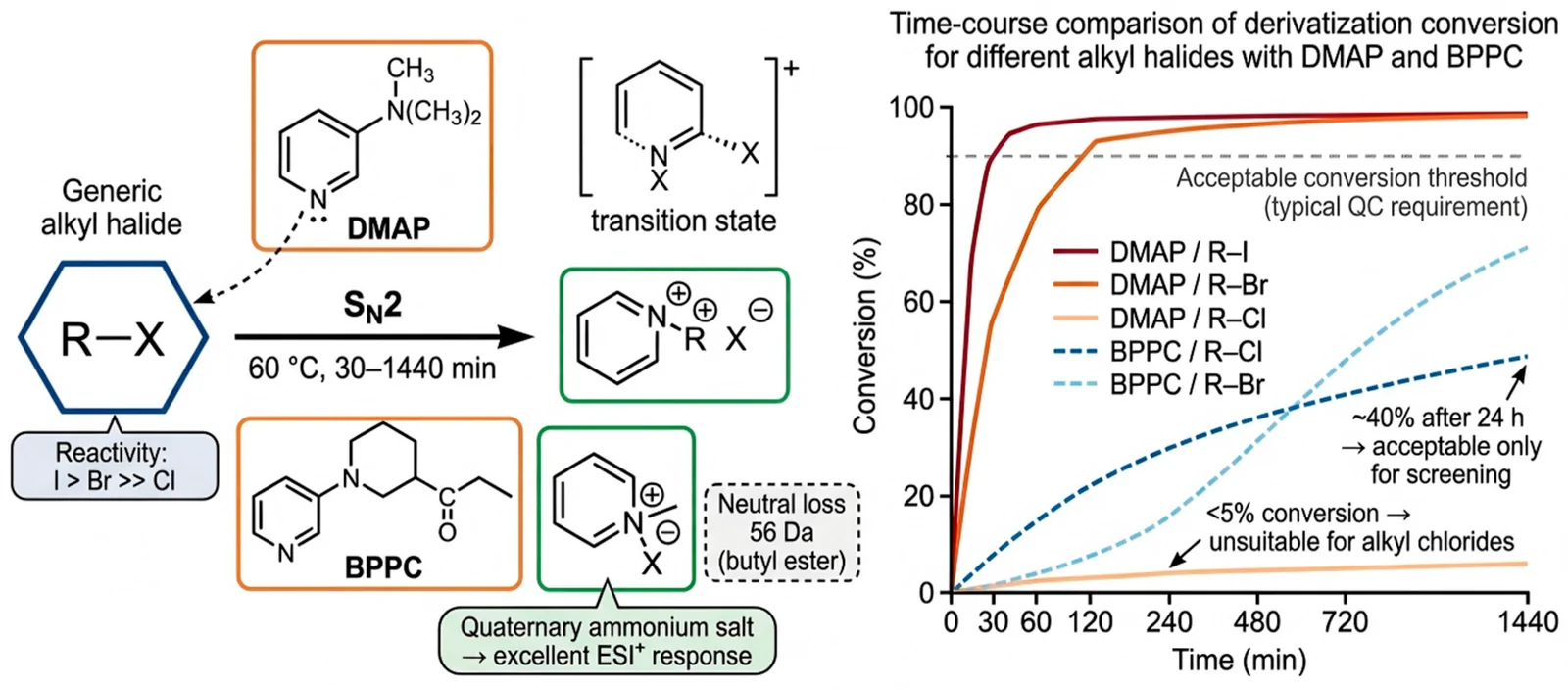
SN_2_ derivatization mechanism and reactivity comparison of alkyl halides. The reaction converts alkyl halides to quaternary ammonium salts; the reactivity order (iodides > bromides ≫ chlorides) dictates the required reaction time and reagent.

The SN_2_ derivatization of alkyl halides with DMAP (for iodides and bromides) or BPPC (for chlorides) is well understood from a mechanistic perspective (Section 3.2). As illustrated in Figure 4, the reaction proceeds via nucleophilic attack of the pyridine nitrogen on the electrophilic carbon, and the reactivity follows the order I > Br ≫ Cl. In practice, however, the most frequently overlooked issue is matrix interference from the API itself. Many active pharmaceutical ingredients contain nucleophilic functional groups such as amines, alcohols, or thiols, which compete with the target GTI for the same derivatization reagent. This competition reduces the effective reagent concentration and leads to lower or more variable conversion yields. A simple diagnostic experiment can reveal such interference: the derivatization yield of the GTI spiked into neat solution is compared with that spiked into the API matrix at its nominal concentration. A recovery deviation exceeding ±15% confirms competitive consumption [30]. Once confirmed, several mitigation strategies are available. Increasing the molar excess of the derivatization reagent from 10-fold to 50-fold often restores acceptable yield. Alternatively, a selective sample cleanup step, such as solid-phase extraction, can remove nucleophilic API components before derivatization. When neither approach is feasible, switching to a less reactive reagent—such as BPPC, which shows fewer side reactions than DMAP—may help, albeit at the cost of longer reaction times [42]. A more fundamental safeguard is the use of an isotopically labeled internal standard (IL-IS) that undergoes the same SN_2_ reaction, as it cancels out yield fluctuations regardless of batch-to-batch variations in API matrix [35]. For routine quality control of known alkyl iodides and bromides, DMAP remains the first choice because of its fast reaction (30 min) and high conversion (>95%). BPPC should be reserved for cases where alkyl chlorides are suspected or where neutral loss scanning (56 Da) is required for selectivity in complex matrices; the 24 h reaction time is acceptable only if the method is automated and an IL-IS is employed. Çeğil et al. developed a UPLC-MS method for dimethyl sulfate in pantoprazole using triethylamine derivatization, achieving an LOD of 1.94 × 10^−7^ mg/mL and recoveries of 96.46–105.98% [58].

### 4.2. Nitroaromatics

Nitroaromatic GTIs require reduction to the corresponding aniline before LC-MS detection. The challenge is not the reduction itself but rather the selectivity of the reducing agent, which may also attack reducible groups present in the API, such as aldehydes, ketones, halogens, or even carbon–carbon double bonds [50]. Diagnosing such interference follows a similar logic as for alkyl halides: the recovery of the aniline derivative from a matrix-spiked sample is compared with that from a neat solution. A significant reduction in recovery suggests that the API contains competing reducible functionalities. Confirmation can be obtained by spiking a surrogate reducible compound, such as 4-nitrobenzaldehyde, and monitoring its reduction products. The choice of reducing system is critical. The Zn/NH_4_COOH combination is the first-line option for most APIs because it operates at room temperature, is fast (30 min), and shows good selectivity for aromatic nitro groups in the presence of many other functionalities [50]. When interference is observed—for example, with APIs containing halogen substituents—sodium dithionite (Na_2_S_2_O_4_) provides a milder alternative, although it generates sulfur-containing by-products (sulfite, thiosulfate) that require solid-phase extraction cleanup to avoid ESI suppression [59]. Catalytic hydrogenation (Pd/C/H_2_) is powerful but non-selective; it should be considered only when the API is completely inert to hydrogenation and the laboratory is equipped for safe hydrogen handling [60]. A systematic comparison of these three reduction systems is summarized in Table 2. Regardless of the system chosen, validation must be performed with multiple API batches, because batch-to-batch variations in API surface chemistry or particle size can affect reduction efficiency. A single spiked recovery experiment is insufficient. Whenever possible, an IL-IS (e.g., a deuterated aniline derivative) should be used to correct for yield variations.

### 4.3. Sulfonyl Chlorides

For sulfonyl chloride GTIs, the dominant problem is not poor MS response but chemical instability. These compounds hydrolyze rapidly in the presence of trace moisture, degrading during sample preparation and chromatography. Derivatization with a primary amine, such as benzylamine, locks the reactive –SO_2_Cl moiety as a stable sulfonamide while simultaneously introducing a protonatable group for efficient ESI^+^ detection [23]. The success of this approach hinges on timing and reaction conditions: the derivatization reagent must be added immediately after dissolving the sample, before any aqueous mobile phase is introduced, and the reaction solvent must be freshly opened anhydrous acetonitrile or dichloromethane. Even under optimized conditions, it is prudent to include a control sample spiked with a surrogate sulfonyl chloride (e.g., benzenesulfonyl chloride) in each analytical batch to monitor derivatization yield. Additionally, the stability of the sulfonamide derivative in the autosampler should be validated for at least 24 h under the anticipated storage conditions (typically 10 °C). Methods that neglect these precautions often fail during transfer between laboratories because of seemingly minor differences in ambient humidity or solvent quality. Thumbar et al. developed an LC-MS/MS method for a genotoxic sulfonyl chloride impurity in topiramate using benzylamine derivatization. The impurity hydrolyzes rapidly in moisture, requiring immediate derivatization after sample dissolution. The method achieved an LOD of 0.072 µg·mL^−1^ and recoveries of 96.8–104.4%, enabling trace-level quantification below 1 ppm [23].

### 4.4. Hydroxylamine

Hydroxylamine is a small, highly polar molecule that shows virtually no retention on reversed-phase columns and negligible ESI response. Derivatization with FMOC-Cl is effective, but FMOC-Cl reacts indiscriminately with any nucleophile, including the API itself when it contains amino or hydroxyl groups [51,62]. To overcome this lack of selectivity, Song and co-workers introduced an elegant sample pretreatment step: the API is dissolved in a suitable solvent, then an anti-solvent is added to precipitate the API, and after centrifugation the supernatant (containing hydroxylamine) is derivatized. This approach was successfully demonstrated for vorinostat and zileuton, two APIs with highly reactive groups that would otherwise consume the derivatization reagent [51]. For analysts facing similar interference, the key lesson is to explore whether the API can be selectively precipitated without co-precipitating the GTI. Hydrophilic GTIs like hydroxylamine are likely to remain in the supernatant. Once the API is removed, the derivatization proceeds smoothly under aqueous conditions at pH 9.5 with borate buffer, forming a bis-FMOC derivative that gives excellent ESI^+^ response. Although the original method used HPLC-UV, the same derivatization is directly transferable to LC-MS/MS, where MRM detection provides even greater selectivity.

A related derivatization strategy for hydroxylamine-type impurities was reported by Shah et al., who employed dansyl chloride to derivatize N,O-dimethylhydroxylamine, a polar genotoxic impurity structurally similar to hydroxylamine, lacking a chromophore and with poor chromatographic retention. The reaction proceeded at 60 °C for 30 min, and the LC-MS method demonstrated linearity from 5 to 60 ppm (relative to 10 mg·mL^−1^ drug substance concentration), with acceptable specificity, accuracy, and repeatability [63]. This work illustrates that dansyl chloride offers an alternative to FMOC-Cl for hydroxylamine-type impurities, particularly when the API matrix does not interfere with the derivatization reaction.

### 4.5. Alcohols and Hydrazines

For some hydroxy or hydrazine GTIs, even conventional ESI-friendly derivatization fails because the derivative still ionizes poorly or because the API matrix causes severe ion suppression. In such cases, elemental tagging with ICP-MS offers an alternative that bypasses ESI-related problems entirely [64]. The iodine atom is then detected by ICP-MS with sub-ppb sensitivity and minimal matrix effects, because the ICP source atomizes the analyte and measures the iodine isotope independently of the organic structure. This strategy is best reserved for GTIs where all ESI-compatible methods have been exhausted and the regulatory limit cannot otherwise be met. The main drawbacks are the requirement for specialized ICP-MS instrumentation, which is not standard in most pharmaceutical QC laboratories, and the limited tolerance of ICP-MS to organic mobile phases (typically ≤20–40% acetonitrile, with oxygen addition to maintain plasma stability). Nevertheless, for challenging analytes such as aliphatic alcohols and hydrazines, elemental tagging has proven to be a powerful last resort.

### 4.6. Epoxides

Epoxides are neutral, poorly ionizable, and prone to hydrolysis. Conventional solution-phase derivatization, for instance with diethyldithiocarbamate, requires heating at 60 °C for 20 min and subsequent cleanup, which is acceptable but not ideal for high-throughput workflows [57,65,66]. A conceptually different approach is gas-phase derivatization via the Meerwein reaction. In this method, ethyl nitrilium ions generated from acetonitrile in an APCI source react instantaneously with epoxides to form detectable adducts [56,66]. The derivatization occurs in the ion source after LC separation, requiring no sample pretreatment, no additional reagents, and no reaction coil. The method is class-selective for epoxides and achieves limits of detection ≤1 ppm in API matrices with excellent accuracy (92–102% recovery) and precision (RSD ≤ 2.2%) [66]. For laboratories equipped with an APCI source, gas-phase derivatization is the preferred choice because of its speed and simplicity. For those lacking APCI capability, solution-phase derivatization with diethyldithiocarbamate remains a practical fallback, although matrix effects and yield consistency must be carefully validated. The gas-phase approach is a striking example of how moving the derivatization from the liquid phase to the gas phase can circumvent the kinetic and band-broadening limitations that plague conventional post-column methods.

Beyond epoxides, gas-phase and solution-phase derivatization strategies have also been applied to other GTI classes. Neşetoğlu and Ünal developed and validated two impurity-specific methods for pharmaceutical products: a GC-MS method for volatile alkylating agents—methyl, ethyl, and isopropyl methanesulfonate—in imatinib mesylate API, and an LC-MS/MS method for 5-(4′-(azidomethyl)-[1,1′-biphenyl]-2-yl)-1H-tetrazole, a polar and thermolabile azido impurity, in valsartan formulations. Both methods demonstrated excellent specificity, linearity (R^2^ > 0.999), accuracy, and precision, with detection limits sufficient to meet ICH M7 regulatory requirements. This work illustrates that the choice between GC-MS and LC-MS/MS for GTI analysis depends on the physicochemical properties of the target impurity—volatility for GC-MS versus thermal stability and polarity for LC-MS/MS [52].

### 4.7. Aldehydes, Ketones, Carboxylic Acids, and Amines

The remaining common GTI classes—aldehydes, ketones, carboxylic acids, and amines—are often detectable by ESI-MS to some extent, but derivatization can improve sensitivity, chromatographic retention, or selectivity. For aldehydes and ketones, 3-nitrophenylhydrazine (3-NPH) has emerged as a versatile reagent. It reacts under mild conditions (room temperature, 30 min) to form stable hydrazone derivatives, and the nitrophenyl group fragments characteristically in ESI-MS/MS, enabling highly selective MRM detection [20]. Compared with the traditional 2,4-dinitrophenylhydrazine (DNPH), 3-NPH gives lower background and better sensitivity. For volatile aldehydes such as hexanal, derivatization with pentafluorobenzylhydroxylamine (PFBHA) followed by GC-ECNI-MS offers excellent sensitivity (LOD 50 pg injected) [31]. For carboxylic acids, 3-NPH is also effective when used with a condensing agent such as a carbodiimide; the resulting 3-nitrophenylhydrazide derivatives give intense signals in ESI^+^ mode [7,9,44]. Valproic acid, for example, has been quantified at LODs as low as 25 nM (approximately 0.006 ppm) using this approach [7]. Before derivatizing a carboxylic acid, however, it is prudent to test the underivatized analyte in ESI^−^ mode; if the sensitivity meets regulatory requirements, derivatization may be unnecessary. For amines, which are inherently ionizable as [M+H]^+^, derivatization is rarely needed for sensitivity but can be valuable for improving chromatographic separation from the API or for adding characteristic MS/MS fragments. FMOC-Cl reacts rapidly (≤5 min) with primary and secondary amines to form stable carbamates that are well retained on reversed-phase columns [23,35]. For small aromatic amines like 2-aminopyridine, using in situ derivatization with hexyl chloroformate produces amide derivatives with improved retention and detection limits below 1 ppm [27]. For N-oxide-containing GTIs, direct LC-MS/MS analysis has been successfully applied without derivatization. A recent impurity profiling study on the anti-NAFLD drug IMM demonstrated that impurity V, an oxidation by-product containing an N-oxygen fragment, could be quantified by HPLC-MS/MS at LODs as low as 0.05 ng/mL, eliminating the need for chemical derivatization [67]. In all these cases, the decision to derivatize should be guided by a simple question: does the underivatized GTI meet the required LOQ in the actual API matrix? Only when the answer is no should derivatization be pursued. For GTIs with sufficient intrinsic ionization efficiency under ESI^+^ conditions, direct LC-MS/MS analysis can be effectively applied without derivatization. Aparna et al. developed a validated LC-MS/MS method for two genotoxic impurities (GTS-STG-1A and GTS-STG-1B) in moxifloxacin hydrochloride API, achieving linear ranges of 0.3863–5.7938 ppm and 0.3786–5.6790 ppm, respectively, with accuracies of 94.6–99.35% and precisions of 1.40–4.30% [65]. This case reaffirms that derivatization is not mandatory when the GTI exhibits adequate ionization efficiency under electrospray ionization.

## 5. Comparison of Pre-Column and Post-Column Derivatization Modes

The position of the derivatization reaction in the analytical workflow directly affects sensitivity, operational complexity, and automation potential. Derivatization can be performed either before chromatographic separation (pre-column) or after separation (post-column). Pre-column derivatization is by far the dominant choice in GTI analysis, whereas post-column derivatization, despite its theoretical advantage of on-line automation, has found very few practical applications. This chapter first compares the principles and applicability of the two modes, then analyses the practical obstacles that post-column derivatization faces in GTI analysis, and finally discusses emerging on-line strategies—including gas-phase ion-molecule reactions and automated pre-column workflows—that offer alternative paths forward. Recent advances in multidimensional chromatographic and mass spectrometric strategies have addressed key challenges in resolving complex pharmaceutical matrices, including co-elution, trace-level detection, and ion suppression, providing a useful context for understanding why post-column derivatization has seen limited adoption in GTI analysis [68].

### 5.1. Pre-Column Derivatization

Pre-column derivatization, in which the derivatization reaction is performed prior to chromatographic separation, is the overwhelmingly dominant mode in GTI analysis due to its operational flexibility and compatibility with established LC-MS workflows [19]. Its popularity stems from three core advantages that align well with the practical constraints of trace impurity analysis.

Flexibility in reaction conditions. Unlike post-column setups, pre-column derivatization imposes no constraints on reaction time, temperature, or solvent composition. Reactions can be carried out at elevated temperatures (e.g., 50–100 °C) for extended periods (from 30 min to 24 h) using non-volatile buffers or purely organic solvents. This flexibility is essential for many GTI derivatizations that exhibit slow kinetics, such as the BPPC-mediated quaternization of alkyl chlorides (24 h at 60 °C) [42]. In the 35+ methods compiled in Table 1, all employ pre-column derivatization.

The pre-column derivatization workflow supports batch processing and high-throughput analysis, enabling parallel sample preparation using 96-well plates or multi-tube racks, followed by sequential injection. Pre-column derivatization can be performed in parallel using 96-well plates or multi-tube racks, followed by sequential injection. This workflow is particularly advantageous for stability studies, batch release testing, or any scenario requiring analysis of dozens to hundreds of samples. Moreover, the reaction can be easily combined with internal standard addition, dilution, filtration, or solid-phase extraction cleanup before injection, further enhancing method robustness [19,20].

Decoupling of reaction and separation. Because the derivatization is completed before the sample enters the LC system, any side products, excess reagent, or matrix components that do not interfere with the reaction can be removed or diluted prior to injection. This reduces the risk of column contamination and ion source fouling—a non-trivial benefit when working with reactive reagents like FMOC-Cl or DMAP.

However, pre-column derivatization is not without limitations. Three drawbacks warrant particular attention. Three drawbacks warrant particular attention. First, additional sample handling steps are introduced an extra offline operation (mixing, heating, possibly cleanup), which increases total analysis time and the risk of human error. For reactions requiring precise timing or anhydrous conditions, even minor deviations can lead to poor reproducibility. This is especially critical for highly moisture-sensitive GTIs such as sulfonyl chlorides, where trace water in the atmosphere or solvent can cause competitive hydrolysis before the derivatization reagent is added.

Risk of incomplete or variable conversion. Because the reaction is performed offline, conversion yield is subject to batch-to-batch variations in temperature, reagent lot, solvent quality, and operator technique. As discussed in Section 2.3, a method that achieves 95% conversion in the hands of the original developer may yield only 70% in a different laboratory if critical parameters are not strictly controlled. The absence of an isotopically labeled internal standard amplifies this risk, as yield fluctuations translate directly into quantification errors.

Potential interference from by-products and excess reagent [69]. Unreacted derivatization reagent or its degradation products (e.g., DMAP dimer, FMOC-OH) can co-elute with the target derivative, causing ion suppression or false-positive signals. For example, a dimeric byproduct of DMAP formed at elevated temperatures produces an intense [M+H]^+^ signal in ESI^+^ mode. When it co-elutes with the derivative, the dimer competes for droplet charges, causing ion suppression. In cases where the dimer’s m/z or CID fragments overlap with those of the derivative, its presence can also generate false-positive MRM signals, either by direct transmission through Q1/Q3 filters or via MRM cross-talk between adjacent transitions. The interference therefore stems not from a single mechanism but from a combination of charge competition in the ESI source and mass filter overlap in the MS analyzer. Detecting its origin requires systematic approaches: infusion of the pure dimer reveals its precursor and product ions; a reagent blank chromatogram identifies its retention time; and injection of a derivatized control standard while monitoring two complementary MRM transitions for the derivative (ion ratio acceptance within ±20%) distinguishes between true analyte signals and dimer-related artifacts.

The formation of such dimeric by-products is a well-recognized issue in DMAP-based derivatization methods and has been studied in the context of method development for trace impurity analysis [34]. While such interferences can be mitigated by optimizing reaction conditions (lower temperature, shorter time) or by post-reaction cleanup, they add an extra layer of method development complexity.

To maximize the reliability of pre-column derivatization methods for GTI analysis, several practical measures should be integrated into routine workflows. First, a derivatized control standard should be included in every analytical batch to monitor conversion consistency; this provides an immediate check for batch-to-batch variations in reaction conditions. Second, during method validation, acceptable ranges for critical parameters—such as temperature (±2 °C), reaction time (±5 min), and reagent concentration (±10%)—must be explicitly defined and subjected to robustness testing. These validation practices are aligned with recommendations from recent method validation guidelines [19,33]. Third, a reagent blank chromatogram is essential to identify by-product peaks and confirm that they do not co-elute with the target derivative; if co-elution proves unavoidable, high-resolution mass spectrometry or an alternative MRM transition ratio check should be employed. Finally, whenever possible, an isotopically labeled internal standard that undergoes the same derivatization reaction should be used, as it effectively cancels out yield fluctuations and provides the most reliable quantification.

In summary, pre-column derivatization remains the most practical and widely adopted mode for GTI analysis due to its flexibility, batch compatibility, and decoupling of reaction from separation. Its limitations—additional handling, variable conversion, and by-product interference—can be managed through rigorous method validation, appropriate internal standards, and careful control of reaction conditions. For the vast majority of GTI applications, the benefits of pre-column derivatization far outweigh its drawbacks.

### 5.2. Post-Column Derivatization

In post-column derivatization, the derivatization reagent is continuously added via a second pump into the LC eluate after the column, and the reaction takes place in a coiled reactor (typically a few meters of tubing maintained at an elevated temperature) before the mixture enters the detector. This mode offers two theoretical advantages over pre-column approaches: full automation (the reaction is performed online, reducing manual intervention) and minimal matrix effects on the derivatization step, since the target analytes have already been separated from most matrix components by the time they reach the reactor [20].

Despite these appealing features, post-column derivatization has found virtually no practical application in GTI analysis due to three fundamental constraints—slow reaction kinetics, extra-column band broadening, and continuous background interference [70].

First, the most prohibitive obstacle is reaction kinetics. Post-column derivatization requires the derivatization reaction to be completed within tens of seconds to a few minutes—the time window defined by the flow rate and the reactor volume. In contrast, virtually all derivatization reactions used for GTIs, including SN_2_ substitutions (e.g., DMAP or BPPC with alkyl halides), esterifications, amidations, and hydrazone formations, require 15–30 min at room temperature to reach acceptable conversion, and many require heating for 30 min to 24 h. Even with elevated temperatures in a post-column reactor coil, the residence time is rarely sufficient for slow-reacting GTIs such as alkyl chlorides [42]. In other words, the same sluggish kinetics that makes pre-column derivatization time-consuming makes post-column derivatization impractical.

Second, an often overlooked hurdle is extra-column band broadening. The reaction coil, especially when long enough to provide adequate residence time, adds significant volume to the post-column flow path. This additional volume degrades chromatographic resolution, which is particularly problematic when a trace GTI derivative must be separated from a large, tailing API peak that elutes nearby. As highlighted by Jones et al., the extra post-column dead volume due to reaction coils causes peak broadening and a loss of separation power, and practitioners of PCD methods largely avoid the use of UHPLC-type column formats because such formats are incompatible with conventional PCD setups [71]. In GTI analysis, where selectivity is already challenged by matrix interference, sacrificing resolution is rarely acceptable.

Third, an obstacle arises from the continuous infusion of derivatization reagent into the mass spectrometer. Unlike pre-column derivatization, where excess reagent can be diluted, removed by SPE, or diverted to waste before the ion source, post-column derivatization forces the reagent and its by-products to flow directly into the MS. This creates a constant chemical background that elevates baseline noise and can cause severe ion suppression. Moreover, matrix components can also suppress the derivatization reaction yield of the target analytes themselves, further complicating the analytical outcome [44]. For trace analysis at the low ppm or ppb levels mandated by ICH M7, even a modest background signal may push the limit of detection above the required threshold.

One potential future direction to overcome the kinetic limitation is microfluidic post-column derivatization, where the extremely short diffusion distance in microchannels dramatically enhances mass transfer. Although this approach could theoretically reduce reaction times from hours to minutes, no application to GTI analysis has been reported to date. Taken together, these three obstacles—slow reaction kinetics, extra-column band broadening, and continuous background interference—explain why conventional liquid-phase post-column derivatization has not been adopted for routine GTI analysis.

### 5.3. Emerging On-Line Strategies: Gas-Phase and Automated Pre-Column Approaches

Given the impracticality of conventional post-column derivatization for GTIs, two alternative on-line strategies have emerged that circumvent its limitations: gas-phase ion-molecule reactions (which occur in the ion source) and automated pre-column derivatization (which uses modern autosamplers to automate the pre-column workflow). These approaches are conceptually distinct from liquid-phase PCD but offer viable paths toward automation and improved throughput [16].

#### 5.3.1. Gas-Phase Derivatization via Ion–Molecule Reactions

Kord and co-workers reported a fundamentally different approach: gas-phase derivatization integrated directly into the ionization source [56,66]. Ethyl nitrilium ions, generated in an atmospheric pressure chemical ionization (APCI) source from acetonitrile, react with epoxides via a Meerwein reaction to form detectable adducts. The entire derivatization occurs instantaneously in the gas phase, requiring no sample pretreatment, no additional reagents, and no reaction coil. This method is class-selective for epoxides and achieves LODs ≤1 ppm in API matrices with excellent accuracy and precision (92–102% recovery, RSD ≤ 2.2%) [66].

Gas-phase derivatization is fundamentally distinct from conventional post-column derivatization, and this distinction is critical to understanding its unique applicability in GTI analysis. Rather than occurring in a liquid-phase reactor downstream of the column, the derivatization reaction takes place directly within the ion source, following nebulization and vaporization of the LC eluate. This seemingly technical difference carries profound practical implications. In conventional liquid-phase PCD, the residence time in the reaction coil is limited to seconds or minutes—far too short for the inherently slow SN_2_ substitutions, esterifications, and hydrazone formations required for most GTIs. Gas-phase derivatization circumvents this kinetic bottleneck entirely, as ion-molecule reactions proceed on a millisecond timescale. Moreover, the absence of a reaction coil eliminates extra-column band broadening, preserving chromatographic resolution that would otherwise be compromised by the additional dead volume of a liquid-phase reactor. Equally importantly, gas-phase derivatization generates reactive species in situ from mobile phase components (e.g., acetonitrile), avoiding the continuous chemical background introduced by externally pumped reagents—a persistent challenge that elevates baseline noise and compromises detection limits in liquid-phase PCD. These differences are not merely operational; they fundamentally determine whether online derivatization is feasible for a given GTI class.

Currently, this method is limited to epoxides and requires an APCI source. However, the concept—moving derivatization from the liquid phase to the gas phase—opens a new paradigm. If similar ion-molecule reactions can be developed for other reactive GTI classes (e.g., aldehydes, aziridines, certain alkylating agents), gas-phase derivatization could become a powerful online screening tool. For now, it remains a specialized but highly elegant solution for epoxide analysis.

#### 5.3.2. Automated Pre-Column Derivatization Using Modern Autosamplers

Many current LC autosamplers can be programmed to mix sample and reagent in a vial, incubate at a controlled temperature for a defined period, and then inject the reaction mixture directly onto the column. This approach automates the pre-column workflow, eliminating manual handling and improving reproducibility. It is not post-column derivatization in the strict sense—the reaction still occurs before separation—but it represents an important step toward on-line operation.

Automated pre-column derivatization has been increasingly integrated into LC-MS workflows for high-throughput GTI screening. For reactions that complete within 30 min at room temperature or with moderate heating (e.g., DMAP derivatization of alkyl iodides and bromides, FMOC-Cl derivatization of hydroxylamine), such automated systems offer a practical compromise between the flexibility of offline derivatization and the convenience of true on-line operation [10]. When coupled with 96-well plate handling, they enable parallel processing of dozens of samples with precise control of reaction parameters.

The main limitation remains reaction kinetics: for slow reactions (e.g., BPPC with alkyl chlorides, 24 h), automated autosampler programming is impractical because it would tie up the instrument for an entire day. In such cases, batch processing in a separate heating block or oven, followed by transfer to the autosampler, remains the preferred approach.

#### 5.3.3. Photochemical Derivatization: A Note of Caution

Online photochemical derivatization uses UV irradiation to induce structural changes (e.g., isomerization, cyclization, or photooxidation) without requiring an exogenous reagent. It has been successfully employed for the LC-MS/MS analysis of aflatoxins and other mycotoxins, where UV light converts poorly ionizable species into more detectable products. However, photochemical derivatization has found virtually no application for GTIs. The key reason is that most GTIs—alkyl halides, nitroaromatics, sulfonyl chlorides, epoxides, and small aldehydes—lack the extended π-conjugation or photosensitive functional groups necessary for efficient photochemical transformation under typical UV irradiation (254 nm or 365 nm). Additionally, photochemical reactions in flow-through reactors often produce complex by-product mixtures that are unpredictable and may co-elute with the derivative, introducing selectivity risks. Thus, while photochemical derivatization is a valuable tool for certain compound classes, it does not address the fundamental ESI-invisibility of most GTIs.

### 5.4. Systematic Comparison of Pre-Column and Post-Column Derivatization

As summarized in Table 3, pre-column and post-column derivatization differ markedly in reaction kinetics, automation feasibility, chromatographic resolution, matrix effects, and regulatory acceptance. In contrast, post-column derivatization, despite its theoretical advantage of online automation, is severely constrained by three fundamental mismatches: first, the short residence time in the reaction coil (seconds to minutes) versus the slow kinetics of most GTI derivatizations (30 min to 24 h); second, extra-column band broadening from the reaction coil, which compromises resolution and precludes UHPLC compatibility; and third, continuous infusion of reagent and byproducts into the MS, which elevates baseline noise, causes ion suppression, and complicates validation [44,70,71].

## 6. Conclusions and Perspectives

### 6.1. Core Consensus: Derivatization as the Most Effective Strategy for “Invisible” GTIs

Derivatization has proven to be the most effective bridge between “undetectable” genotoxic impurities and sensitive LC-MS analysis. The core consensus emerging from over a decade of research can be summarized as follows. Quantitatively, derivatization typically enhances ESI-MS detectability by 50- to 1000-fold when comparing peak areas or signal-to-noise ratios of the derivative versus the underivatized analyte under identical LC-MS/MS conditions. This enhancement range, compiled from the studies reviewed in Table 1, is reflected in the corresponding improvements in limits of detection (LOD) and quantification (LOQ). The fold-increase is therefore not a single universal metric but is reported here as a conservative estimate based on the most commonly compared parameter (peak area) in the original references; where S/N was reported, the enhancement was similar. In all cases, the enhancement is sufficient to bring LODs consistently below the 1-ppm threshold required for routine GTI control. This order-of-magnitude improvement is the operational definition of the “undetectable to sensitive detection” transition highlighted in the title.

First, derivatization addresses the fundamental limitation of electrospray ionization—namely, the inability to ionize neutral, non-basic, or non-acidic small molecules. By introducing permanent charges (e.g., quaternary ammonium salts via SN_2_ with DMAP or BPPC) or protonatable groups (e.g., amino groups after reduction of nitroaromatics), derivatization transforms poorly responsive GTIs into highly detectable derivatives. Second, beyond simple detectability, derivatization can be designed to embed MS/MS information carriers (e.g., the neutral loss of 56 Da from BPPC), thereby enhancing selectivity and enabling screening of unknown mixtures. Third, derivatization can also stabilize reactive GTIs (e.g., sulfonyl chlorides locked as sulfonamides) and improve chromatographic retention of highly polar analytes (e.g., hydroxylamine derivatized with FMOC-Cl).

However, the successful application of derivatization requires systematic evaluation of three critical metrics: conversion yield (a minimum of 70% is generally recommended for quantitative methods), reproducibility (including robustness testing across different conditions and laboratories), and by-product interference (via reagent blanks and peak purity checks). Methods that fail to address these metrics are unlikely to transfer reliably to routine quality control.

**Figure 5:**
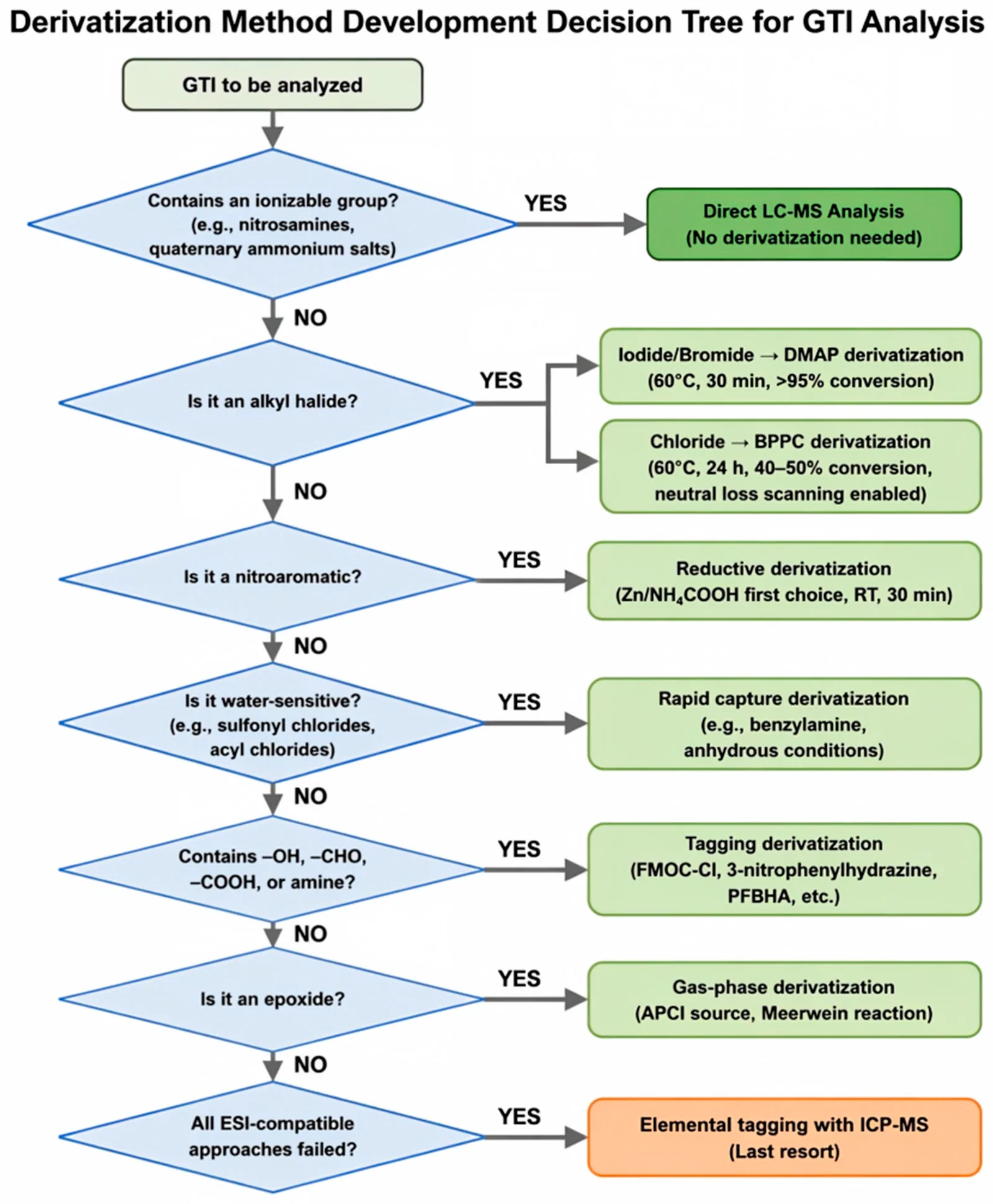
Derivatization Method Development Decision Tree for Genotoxic Impurity (GTI) Analysis.

To assist analysts in navigating the various derivatization options, Figure 5 presents a practical decision tree for selecting a derivatization strategy based on the GTI’s functional group and physicochemical properties. The workflow proceeds as follows: (i) if the GTI contains an ionizable group (e.g., nitrosamines, quaternary ammonium salts), direct LC-MS analysis is attempted first; (ii) if not, the presence of a halogen directs the choice between DMAP (for iodides/bromides, fast and high conversion) and BPPC (for chlorides, slower but enables neutral-loss MS/MS screening); (iii) for nitroaromatics, reductive derivatization (preferably Zn/NH_4_COOH) is recommended; (iv) water-sensitive GTIs (e.g., sulfonyl chlorides, acyl chlorides, chloroformates) require rapid capture derivatization with an appropriate nucleophile; (v) for other polar functional groups (-OH, -CHO, -COOH, amines), universal tags such as FMOC-Cl or 3-nitrophenylhydrazine are effective; (vi) for epoxides, gas-phase derivatization via the Meerwein reaction in an APCI source offers a selective and rapid alternative; and (vii) if all ESI-compatible approaches fail, elemental tagging with ICP-MS serves as a powerful last resort. This decision tree consolidates the strategies discussed in Section 4.1, Section 4.2, Section 4.3, Section 4.4, Section 4.5, Section 4.6 and Section 4.7 and provides a starting point for method development in routine quality control.

### 6.2. Current Bottlenecks

Despite the proven utility of derivatization, several persistent bottlenecks limit its broader application and robustness in GTI analysis. These challenges differ in their practical frequency and severity, and a hierarchical consideration is instructive for method development.

Method transfer difficulties are among the most frequently encountered obstacles in routine quality control. Derivatization-based methods are notoriously sensitive to seemingly minor changes in solvent brand, ambient temperature, reaction time, or laboratory humidity. The dimerization of DMAP at elevated temperatures, the hydrolysis of sulfonyl chlorides in the presence of trace water, and the oxidation of DMAP by peroxides in acetonitrile are well-documented yet under-reported pitfalls. Consequently, a method that passes validation in one laboratory may fail during transfer to another QC site.

API matrix interference represents a more critical concern, as the API itself often competes for the derivatization reagent or reducing agent, particularly when the API contains nucleophilic or reducible groups. This competition is rarely evaluated in published methods; a simple spiked recovery experiment is insufficient to detect batch-to-batch variations in API reactivity. In the absence of an isotopically labeled internal standard, such matrix effects can cause severe under- or overestimation of GTI levels.

Poor selectivity of derivatization reagents is another significant limitation. Many widely used reagents—notably FMOC-Cl—lack selectivity and react with any nucleophile present in the API or matrix. While clever sample pretreatment (e.g., API precipitation) can mitigate interference, such steps add complexity and may co-precipitate the GTI if it is lipophilic. Similarly, reducing agents for nitroaromatics (Zn/NH_4_COOH, Pd/C/H_2_, Na_2_S_2_O_4_) can attack other reducible functional groups in the API, leading to variable yields and false negatives.

Slow reaction kinetics is a more fundamental but comparatively less frequent challenge in routine practice, as many GTIs react rapidly under optimized conditions. However, for less reactive electrophiles such as alkyl chlorides, even 24 h with BPPC yields only 40–50% conversion. Such prolonged reaction times are incompatible with high-throughput workflows and delay batch release in QC laboratories.

Despite the proliferation of derivatization methods for GTIs, the relative performance of different reducing agents and labeling reagents in complex API matrices lacks systematic quantitative comparison and cross-validation. For instance, DMAP [42] and BPPC [42] have been individually reported for alkyl halide analysis, but their tolerance differences toward nucleophilic APIs have never been directly compared under identical conditions. Similarly, the matrix interference behaviors of Zn/NH_4_COOH [50] and Na_2_S_2_O_4_ [61] in nitroaromatic reduction lack parallel evaluation. This data gap forces analysts to rely on trial-and-error during method development rather than evidence-based reagent selection, increasing development timelines and the risk of failure. We recommend that future studies prioritize systematic matrix effect comparison experiments, quantifying recovery deviations, ion suppression severity, and batch-to-batch robustness for mainstream derivatization strategies across representative API matrices, thereby establishing a decision-support database for reagent selection.

### 6.3. Future Directions

Future developments in derivatization-based GTI analysis should be guided not only by scientific promise but also by practical feasibility and regulatory acceptance. The following directions are therefore discussed in order of their technical maturity and near-term applicability, ranging from readily implementable strategies to longer-term exploratory frontiers. Intelligent photochemical/stimuli-responsive derivatization and microfluidic online hyphenation represent emerging frontiers in GTI analysis. Although their application in this field is still in its infancy and published examples remain scarce, these approaches hold substantial promise for addressing current bottlenecks in reaction kinetics and automation, as evidenced by recent advances in adjacent fields—including isotope-coded photochemical derivatization for LC-MS metabolomics [73], microfluidic chip-MS platforms with integrated online derivatization [74], and pillar array micromixers for on-chip post-column derivatization—but remain largely conceptual for GTI analysis and are accordingly addressed within the longer-term perspectives [75]. Accordingly, they are highlighted here as key directions for future research. Addressing the above bottlenecks will require innovation across multiple fronts, from reagent design to workflow automation and green chemistry principles.

#### 6.3.1. High-Throughput Derivatization

Among the emerging strategies, automated pre-column derivatization using modern programmable autosamplers represents the most mature and readily implementable approach. The adoption of 96-well plate formats and automated liquid handling workstations can dramatically increase throughput. Derivatization reactions can be performed in parallel, with precise control of temperature, mixing, and timing. Automated pre-column derivatization using modern autosamplers (with programmable mixing and heating) is already feasible for reactions that complete within 30 min at room temperature. For slower reactions, batch processing in 96-well plates followed by sequential injection remains a practical approach. The key enabler is the standardization of reaction conditions across different GTIs—either by developing universal derivatization cocktails or by using automated systems to execute different programs for each sample. Given the widespread availability of programmable autosamplers in QC laboratories, this automation strategy offers the most immediate pathway to improving inter-laboratory reproducibility and reducing operator-dependent variability, especially for fast-reacting GTIs such as alkyl iodides and bromides. Given its compatibility with existing regulatory frameworks—ICH Q2(R2) validation requirements are equally applicable to automated workflows—this strategy offers the most immediate pathway to enhancing method robustness in routine QC laboratories.

#### 6.3.2. Smart Reagent Design

The evolution from DMAP to BPPC illustrates the potential of embedding MS/MS information into reagent structures. The design of multifunctional derivatization reagents—incorporating MS/MS information carriers, isotope labels, or cleavable linkers—represents a mid-term research frontier. Future smart reagents may incorporate multiple functional modules:

Multi-tag reagents combining a quaternary ammonium group (for ESI response), a labile side chain (for characteristic neutral loss), and a stable isotope label (for isotope dilution quantification) in a single molecule.

Cleavable tags with photocleavable or enzymatically cleavable linkers that allow the tag to be released before MS detection, reducing background noise and improving signal-to-noise ratios. A representative example from proteomics is the photocleavable isotope-coded affinity tag (PC-ICAT), which consists of a biotin affinity handle, a photocleavable o-nitrobenzyl linker, and a mass tag. Upon UV irradiation (365 nm), the linker cleaves, releasing the mass tag (with analyte attached) from the biotin handle, thereby reducing background interference. Although such designs have not yet been reported for GTI derivatization, they offer a promising direction for future reagent development.

Stimuli-responsive reagents that react only under specific conditions (e.g., pH, light, or temperature), thereby improving selectivity. For example, boronate esters for cis-diols or reversible imine bonds could be designed to target specific GTI subclasses.

Ultra-selective reagents for challenging GTIs like hydroxylamine. While FMOC-Cl is non-selective, reagents such as salicylaldehyde (forming a stable oxime) offer higher selectivity but lack MS sensitivity. Future designs could combine a selective capturing group with an MS-enhancing tag.

A recent example of smart reagent design for non-targeted screening was reported by Zhang et al., who developed two complementary derivatization reagents—p-methoxyaniline and p-methoxybenzoyl-β-alaninamide—for the identification of DNA direct reactive impurities (DDRIs). The dual-reagent strategy provided broader functional group coverage (alkylating agents, acylating agents, and epoxides), complementary chromatographic retention, and a 3- to 5-fold sensitivity enhancement through product ion filtering of reporter groups. This approach successfully identified unexpected methylated and ethylated isoquinolinesulfonates in fasudil, illustrating how next-generation reagents can enable non-targeted impurity screening beyond conventional structure-based predictions [3]. Among the remaining challenges, alkyl chlorides and alkyl sulfonates stand out as the most recalcitrant due to their poor leaving-group ability, which necessitates either extremely prolonged reaction times or unacceptably low conversion. Future reagent engineering should therefore prioritize highly nucleophilic scaffolds that can achieve ≥70% conversion within a few hours, while retaining a diagnostic MS/MS fragmentation pattern for selective detection. Similarly, moisture-sensitive GTIs such as sulfonyl chlorides require rapid “capture-and-stabilize” reagents that react in anhydrous media to form stable, ESI-friendly derivatives, ideally in a single step without cumbersome solvent switching. However, it should be recognized that the regulatory acceptance of novel reagents requires extensive validation—including demonstration of derivatization consistency across multiple API batches and laboratories—which inherently limits the speed at which new reagents can be adopted in QC settings.

#### 6.3.3. Online SPE-Derivatization-LC-MS Integration

Fully automated sample-to-result workflows can be achieved by coupling online solid-phase extraction (SPE) with derivatization and LC-MS. In such a system, the sample is loaded onto an SPE column to remove matrix interferences, the eluate is mixed with the derivatization reagent in a reaction coil (or on a microfluidic chip), and the derivatized product is transferred directly to the analytical column. This approach minimizes manual handling, reduces human error, and enables rapid analysis of unstable GTIs that must be derivatized immediately after capture. While technically attractive, the practical realization faces substantial hurdles, including the need for robust interfaces, compatibility with diverse reaction conditions, and validation under GMP-compliant environments. While post-column derivatization remains impractical for most GTIs due to slow kinetics, online pre-column derivatization (using an autosampler as a reaction vessel) is a more realistic near-term goal.

Microfluidics offers a complementary platform. In microchannels, the diffusion distance is extremely short, and mass transfer efficiency is orders of magnitude higher than in conventional reaction vessels. A reaction that takes hours in a vial may be completed in minutes on a microfluidic chip. However, at present, no microfluidic derivatization system has been reported for GTI analysis, and significant engineering challenges—such as chip fouling, pressure tolerance, and reliable coupling to conventional LC-MS—remain unresolved. Thus, while microfluidics merits active exploration as a research direction, its translation to routine QC is unlikely in the near term.

#### 6.3.4. Green Derivatization

Green chemistry principles should inform method development across all stages. With the growing emphasis on green analytical chemistry, derivatization methods should be redesigned to minimize environmental impact:

Aqueous-phase reactions. Many derivatization reactions (e.g., hydrazone formation with aldehydes/ketones, certain reductions) can be performed in water without organic solvents.

Bio-based renewable solvents. When organic solvents are necessary, ethanol, ethyl acetate, or other bio-based solvents can replace conventional petroleum-based solvents (acetonitrile, chloroform).

Low-toxicity reagents. Replace hazardous reagents with safer alternatives. For example, DNPH has toxicity concerns; less toxic phenylhydrazine derivatives could be explored. The environmental fate of FMOC-OH, the hydrolysis product of FMOC-Cl, remains poorly characterized; structurally similar but more environmentally friendly alternatives may be designed.

Minimization of reagent amounts. As column inner diameters decrease and MS sensitivity improves, sample and reagent volumes can be scaled down from milliliters to microliters. This reduces waste generation and reagent costs, and is complementary to high-throughput and microfluidic approaches. These considerations, while not directly tied to regulatory compliance, are increasingly relevant to industrial sustainability goals and are likely to influence method selection in the coming years.

#### 6.3.5. Systematic Evaluation of Matrix Tolerance

A critical unmet need in the field is the establishment of standardized protocols for evaluating and comparing the matrix tolerance of different derivatization strategies. Current literature predominantly reports method performance in a single API matrix, making cross-method comparisons virtually impossible. This fragmented approach hinders evidence-based reagent selection and leads to redundant method development across the industry. To overcome this barrier, we propose a systematic comparative framework. This entails constructing a reference panel of representative APIs with diverse nucleophilic and reducible functional groups, subjecting mainstream derivatization reagents (e.g., DMAP, BPPC, FMOC-Cl, Zn/NH_4_COOH, Na_2_S_2_O_4_) to standardized evaluation against this panel, and mandating the routine reporting of recovery deviations, ion suppression rates, and inter-batch precision. A database compiled from such standardized data would serve as a decision-support tool, accelerating method development and strengthening the reliability of GTI control in regulatory QC settings. Given the diversity of commercial APIs and the lack of publicly available comparative data, this systematic effort is arguably more urgent than the development of yet another single-reagent method for a specific impurity. This effort, while methodologically straightforward, would yield a publicly accessible database with long-term value for the entire field, arguably surpassing the impact of isolated new-reagent reports.

### 6.4. Concluding Remarks

From “undetectable” to “sensitive detection”, derivatization technology has evolved from case-by-case exploration to systematic strategy in GTI analysis. The field now has a solid foundation: functional-group-specific protocols, a growing library of reagents, and a clear understanding of the trade-offs between reactivity, selectivity, and MS compatibility. However, the transition from academic proof-of-concept to robust QC methods remains challenging. Current bottlenecks—slow reaction kinetics for alkyl chlorides and sulfonates, poor reagent selectivity for polar GTIs, API matrix interference, and method transfer difficulties—demand targeted solutions. Among these, the lack of systematic matrix-tolerance data and the limited availability of fast, high-conversion reagents for less reactive electrophiles are the most pressing obstacles. The future lies in high-throughput automation, smart reagent design that embeds MS/MS information, online integration with SPE and microfluidics, and green chemistry principles. By addressing the current bottlenecks—slow kinetics, poor selectivity, API interference, and method transfer difficulties—derivatization will continue to play an indispensable role in ensuring the safety of pharmaceuticals against genotoxic impurities.

## Figures and Tables

**Table 1:** Representative derivatization methods for genotoxic impurities by functional group.

Functional Group	Representative GTI	Derivatization Reagent	Reaction Conditions	Detection	LOD (ppm)	Recovery (%)	RSD (%)	Ref.
Alkyl halide	Methyl iodide	DMAP	60 °C, 30 min, acetonitrile	LC-MS/MS	<0.01	>95	<5	[30]
Alkyl halide	Ethyl bromide	DMAP	60 °C, 30 min, ACN	LC-MS/MS	<0.01	>95	<5	[30]
Alkyl halide	Methyl chloride	BPPC	60 °C, 24 h, ACN/NH_3_	LC-MS/MS (NLS)	~1.0	40–50	<10	[42]
Alkyl halide	Benzyl chloride	BPPC	60 °C, 24 h, ACN/NH_3_	LC-MS/MS (NLS)	~1.0	40–50	<10	[42]
Alkyl halide	Dimethyl sulfate	Triethylamine	RT, 30 min, ACN	UPLC-MS (SIR)	1.94 × 10^−7^	96.5–106.0	<5	[42]
Alkyl halide	Alkyl iodides/bromides (general)	DMAP/BPPC	CE-MS with electrokinetic injection	CE-MS	<1.0	85–115	<10	[48]
Alkyl sulfonate	Alkyl sulfonates (general)	BPPC	60 °C, 24 h, ACN/NH_3_	LC-MS/MS (NLS)	~1.0	NR	NR	[42]
Alkyl sulfonate	Ethyl/propyl/isopropyl sulfonates	Trimethylamine	RT, 30 min, ACN	HILIC-LC/MS	0.2	>85	0.4–4	[49]
Alkyl sulfonate	Methyl sulfonates	Triethylamine	RT, 30 min, ACN	HILIC-LC/MS	0.2	>85	0.4–4	[49]
Nitroaromatic	Nitrobenzene	Zn dust/ammonium formate	Room temperature, 30 min, methanol/water	LC-MS/MS	<0.5	NR	NR	[50]
Nitroaromatic	2-Nitrotoluene	Zn dust/ammonium formate	RT, 30 min, MeOH/H_2_O	LC-MS/MS	<0.2	106–107	3.4–5.4	[50]
Nitroaromatic	3-Nitrotoluene	Zn dust/ammonium formate	RT, 30 min, MeOH/H_2_O	LC-MS/MS	<0.2	103–107	3.4–4.5	[50]
Nitroaromatic	4-Nitrotoluene	Zn dust/ammonium formate	RT, 30 min, MeOH/H_2_O	LC-MS/MS	<0.2	117–119	2.8–4.6	[50]
Nitroaromatic	4-Nitroanisole	Zn dust/ammonium formate	RT, 30 min, MeOH/H_2_O	LC-MS/MS	<0.2	107–108	3.3–3.5	[50]
Nitroaromatic	Methyl 2-(bromomethyl)-6-nitrobenzoate	Zn dust/ammonium formate	RT, 30 min, MeOH/H_2_O	LC-MS/MS	<0.002	80–85	2.1–3.6	[50]
Sulfonyl chloride	Benzenesulfonyl chloride	Benzylamine	50 °C, 30 min, anhydrous acetonitrile	LC-MS/MS	NR	98 (dry solvent)/62 (wet solvent)	NR	[23]
Sulfonyl chloride	Topiramate sulfonyl chloride impurity	Benzylamine	50 °C, 30 min, 1,4-dioxane	LC-MS/MS (MRM)	0.072	96.8–104.4	<5	[23]
Hydroxylamine	Hydroxylamine (NH2OH)	FMOC-Cl	Room temperature, pH 9.5, aqueous	LC-MS/MS	0.33	NR	NR	[51]
Hydrazine	Methyl hydrazine carboxylate	Benzaldehyde	RT, 30 min	LC-MS	1.2	83.7–90.3	3.5	[21]
Hydrazine	1,1-Dimethylhydrazine	Salicylaldehyde	RT, 30 min	LC-MS/MS	0.8	90–102	<6	[17]
Azide	5-(4′-(azidomethyl)-[1,1′-biphenyl]-2-yl)-1H-tetrazole	Direct analysis (no derivatization)	NR	LC-MS/MS	NR	93.4–101.7	0.85–5.90	[52]
Carbamate	Carbaryl	QuEChERS	NR	UHPLC-MS/MS	0.0008	87.5–102.0	<10	[53]
Aziridine	Aziridine	PFBCl (2,3,4,5,6-pentafluorobenzoyl chloride)	SPME, 40 °C, 30 min	GC-MS	NR	95.6–102.4	2.6–8.5	[54]
Pyridinium salt	4-Dimethylaminopyridine	Direct analysis	NR	LC-MS/MS	0.025 (LOQ)	94.0–103.0	1.9–4.9	[34]
Aromatic amine	2-Aminopyridine	Hexyl chloroformate (in situ derivatization)	NR	LC-MS	<1	NR	NR	[45]
Aldehyde	Hexanal	PFBHA (pentafluorobenzylhydroxylamine)	RT, 60 min	GC-ECNI-MS	0.005	NR	NR	[55]
Aldehyde	(S)-tert-Butyl-3-oxo-1-phenylpropylcarbamate (TBC)	2,4-Dinitrophenylhydrazine	RT, 30 min, HCl	LC-MS	0.1	97.4–101.7	3.1	[21]
Carboxylic acid	Valproic acid	3-Nitrophenylhydrazine	RT, 30 min	LC-MS/MS	0.006	85–108	<10	[47]
Epoxide	Propylene oxide	APCI source (Meerwein reaction)	Gas-phase (online)	LC-APCI-MS/MS	≤1.0	92–102	≤2.2	[56]
Epoxide	Epichlorohydrin	Sodium diethyldithiocarbamate	60 °C, 20 min	LC-MS/MS	0.05	90–98	<5	[57]
Acid chloride	Ethyl chloroformate	Aniline	RT, 30 min, pyridine/CCl_4_	LC-MS	10.0	99.4–99.9	4.8	[21]
Alkyl hydrazine carbamate	Methyl-2-(2-chloro-1-iminomethyl) hydrazine carboxylate	Dimethylamine	RT, 30 min	LC-MS	1.2	84.8–92.8	3.1	[21]

Abbreviations: DMAP, 4-dimethylaminopyridine; BPPC, butyl 1-(pyridin-4-yl)piperidine-4-carboxylate; FMOC-Cl, 9-fluorenylmethyl chloroformate; PFBCl, pentafluorobenzoyl chloride; PFBHA, pentafluorobenzylhydroxylamine; SPME, solid-phase microextraction; LOQ, limit of quantification; RT, room temperature; NR, not reported; NLS, neutral loss scanning; MRM, multiple reaction monitoring; ACN, acetonitrile; HILIC, hydrophilic interaction liquid chromatography; CE, capillary electrophoresis; UPLC, ultra-performance liquid chromatography; SIR, selected ion recording; GC-MS, gas chromatography-mass spectrometry; APCI, atmospheric pressure chemical ionization; QuEChERS, Quick, Easy, Cheap, Effective, Rugged, and Safe. Values in bold indicate rows added in this expanded table. All LOD values are converted to ppm (μg·g^−1^) assuming an injection volume of 10 μL and a final sample concentration of 1 mg/mL, unless otherwise specified. Due to differences in API matrices and instrumentation, direct cross-comparison should be made with caution.

**Table 2:** Comparison of reduction systems for nitroaromatic GTI derivatization.

Parameter	Zn/NH_4_COOH	Pd/C + H_2_ (Catalytic Hydrogenation)	Na_2_S_2_O_4_ (Sodium Dithionite)	Ref.
Reaction conditions	Room temperature, 30 min, methanol/water	1–4 bar H_2_, 25–60 °C, 2–24 h, methanol or ethyl acetate	50–70 °C, 30–60 min, aqueous buffer (pH 6–7)	[50,60,61]
Typical conversion	High (>90%)	>95% (may vary with catalyst loading)	79–95% (substrate-dependent)	[50,60]
Selectivity for -NO_2_	Moderate (may reduce aldehydes, ketones, halogens)	Low (significant risk of dehalogenation when API contains halogens)	Moderate (may reduce azo, imine, quinone groups; less effective for sterically hindered –NO_2_)	[61]
Main advantage	Mild conditions, fast, simple workup	High throughput, catalyst recyclable, green (H_2_O byproduct)	Mild, inexpensive, compatible with aqueous matrices	[50,60]
Main limitation	Zn dust residue; potential API interference	Specialized equipment required (H_2_ source, pressure reactor); side reactions (dehalogenation)	May generate sulfur-containing byproducts (sulfite, sulfate, thiosulfate)	[50,60,61]
Incompatible API functional groups	Halogens, carbonyls (aldehydes, ketones)	Halogens (dehalogenation), C=C, C≡C	Azo, imine, quinone	[61]
LC-MS compatibility	Good (reagent residue removable by filtration)	Good (catalyst removal required)	Fair (sulfur compounds may suppress ESI; cleanup recommended)	[50,60]
Practical feasibility	High (standard bench equipment)	Medium (requires H_2_ handling and safety measures)	Medium (requires pH control, byproduct cleanup)	[50,60]

**Table 3:** Systematic comparison of pre-column and post-column derivatization approaches for genotoxic impurity analysis.

Dimension	Pre-Column Derivatization	Post-Column Derivatization	Ref.
Reaction kinetics and conditions	Flexible; 30 min to 24 h at RT-100 °C; compatible with strong nucleophiles, buffers, and non-volatile reagents; slow kinetics (e.g., BPPC with alkyl chlorides, 24 h, 60 °C) is acceptable	Extremely constrained; reaction must complete within seconds to ≤2 min (residence time in reaction coil); only ultra-fast reactions feasible; completely unsuitable for SN2, esterification, amidation, or hydrazone formation	[42,70]
Automation and throughput	Offline or automated via programmable autosamplers; 96-well plate parallel processing for fast reactions (≤30 min); batch sizes of 96–384 samples per run	Fully online and theoretically automated; continuous operation; however, reactor maintenance (coil clogging, bubble formation) reduces practical throughput	[19,20,71]
Chromatographic resolution	Unaffected; derivatization completed before injection; fully compatible with UHPLC (sub-2 µm columns) and high-resolution separations	Severely degraded; reaction coil adds 50–500 µL dead volume, causing peak broadening and loss of resolution; incompatible with UHPLC; practically limited to conventional HPLC	[19,71]
Matrix effects and MS compatibility	Excess reagent and byproducts can be removed by dilution, SPE, or protein precipitation before injection; matrix components do not enter the reaction step	Reagent and byproducts continuously infused into MS; elevated baseline noise (often 3–10× higher); significant ion suppression (typically 20–60% signal loss); severe source fouling	[20,38,44,70]
Method robustness and regulatory acceptance	Well-established; fully compatible with ICH Q2(R2) validation; IL-IS corrects yield fluctuations; robustness testing (temperature ±2 °C, time ±5 min) is standard practice	Poor reproducibility due to variable kinetics and continuous background; no IL-IS correction for on-the-fly reaction yield; rarely validated under regulatory guidelines; minimal regulatory acceptance	[33,34]
Sample throughput and reagent consumption	Moderate-high; reagent consumption per sample (typically 50–200 µL per reaction); scalable to high-density formats (384-well plates)	Continuous reagent consumption (1–5 mL/min); higher operational cost; reagent stability in pump reservoir becomes a critical issue over long runs	[20]
Method development complexity	Moderate; optimization of temperature, time, reagent concentration, and quenching/cleanup steps required; troubleshooting is straightforward	High; requires optimization of coil dimensions, flow rates, reaction temperature, and reagent concentration; complex multi-pump synchronization; difficult to troubleshoot	[19,42,70,71]
Suitability for unstable GTIs	Rapid capture possible (e.g., immediate derivatization of sulfonyl chlorides in anhydrous media); no pre-column degradation risk	Unstable GTIs may degrade during LC separation before reaching the reactor; no immediate stabilization	[23,70]
Derivatization yield control	Full yield monitoring possible via IL-IS or derivatized control standards; yield can be quantitatively determined by pure standard comparison or difference method	Yield cannot be directly measured or verified batch-to-batch; no independent yield assessment; matrix components may suppress the reaction itself	[21,33,44]
Byproduct management	Excess reagent and byproducts can be removed (SPE, dilution, filtration) before injection; reagent blank chromatograms identify interference peaks	Byproducts and excess reagent forced into MS; cannot be removed online; cause constant background and potential false-positive MRM signals	[44]
Applicability to specific GTI classes	Universal	Only suitable for inherently fast reactions; limited to gas-phase Meerwein reaction for epoxides; virtually no published application for other GTI classes	[42,51,56,66,72]
Practical applicability across GTI classes	Widely adopted in pharmaceutical QC	Very limited; virtually no practical adoption for routine GTI analysis; restricted to specialized applications (epoxides with APCI)	[42,50,51,56,66]
Merits	Flexible reaction design; high and consistent conversion achievable; no chromatography compromise; full regulatory acceptance; IL-IS compatible; excess reagent can be removed	Theoretical full automation; minimal manual handling; conceptually elegant; gas-phase variant (APCI) avoids liquid-phase kinetics and band-broadening limitations	[20,66]
Limitations	Additional offline sample handling; risk of conversion variability between batches; byproduct interference if not properly controlled	Slow kinetics mismatch for most GTIs; band broadening; continuous background and ion suppression; difficult to validate; poor regulatory acceptance; high method development complexity	[34,44,69,70,71]

Abbreviations: APCI, atmospheric pressure chemical ionization; BPPC, butyl 1-(pyridin-4-yl)piperidine-4-carboxylate; GTI, genotoxic impurity; IL-IS, isotopically labeled internal standard; MRM, multiple reaction monitoring; MS, mass spectrometry; RT, room temperature; SPE, solid-phase extraction; UHPLC, ultra-high-performance liquid chromatography.

## Data Availability

No new data were created or analyzed in this study. Data sharing is not applicable to this article.

## Abbreviations

The following abbreviations are used in this manuscript:

API	active pharmaceutical ingredient
APCI	atmospheric pressure chemical ionization
BPPC	butyl 1-(pyridin-4-yl)piperidine-4-carboxylate
CE	capillary electrophoresis
CID	collision-induced dissociation
DDRIs	DNA direct reactive impurities
DMAP	4-dimethylaminopyridine
DNPH	2,4-dinitrophenylhydrazine
DIA	data-independent acquisition
ESI	electrospray ionization
FMOC-Cl	9-fluorenylmethyl chloroformate
FT-ICR	Fourier transform ion cyclotron resonance
GC-ECNI-MS	gas chromatography-electron capture negative ionization-mass spectrometry
GTI	genotoxic impurity
HILIC	hydrophilic interaction liquid chromatography
HRMS	high-resolution mass spectrometry
ICP-MS	inductively coupled plasma mass spectrometry
IL-IS	isotopically labeled internal standard
LC-MS	liquid chromatography-mass spectrometry
LC-MS/MS	liquid chromatography-tandem mass spectrometry
LOD	limit of detection
LOQ	limit of quantification
MRM	multiple reaction monitoring
NMR	nuclear Magnetic Resonance
NLS	neutral loss scanning
3-NPH	3-nitrophenylhydrazine
PCD	post-column derivatization
PFBCl	pentafluorobenzoyl chloride
PFBHA	pentafluorobenzylhydroxylamine
QuEChERS	Quick, Easy, Cheap, Effective, Rugged, and Safe
RSD	relative standard deviation
SIR	selected ion recording
SN_2_	bimolecular nucleophilic substitution
SPE	solid-phase extraction
SPME	solid-phase microextraction
TEA	triethylamine
UPLC	ultra-performance liquid chromatography
